# A systematic review of the diversity and virulence correlates of metastrongyle lungworms in marine mammals

**DOI:** 10.1017/S0031182023001014

**Published:** 2023-11

**Authors:** Jared R. Fischbach, Mauricio Seguel

**Affiliations:** 1 College of Biological Sciences, University of Guelph, Guelph, ON, Canada; 2 Department of Pathobiology, Ontario Veterinary College, University of Guelph, Guelph, ON, Canada

**Keywords:** lungworms, marine mammals, metastrongyles, parasites, pathogenicity, virulence

## Abstract

Metastrongyle lungworms could be particularly detrimental for diving animals such as marine mammals; however, little is known of the drivers of pathogenic host–parasite relationships in this group. This systematic review analysed the diversity of metastrongyles in marine mammals and the host and parasite traits associated with virulence. There have been at least 40 species of metastrongyles described in 66 species of marine mammals. After penalization for study biases, *Halocercus hyperoodoni*, *Otostrongylus circumlitus*, *Parafilaroides gymnurus*, *Halocercus brasiliensis* and *Stenurus minor* were the metastrongyles with the widest host range. Most studies (80.12%, *n* = 133/166) reported that metastrongyles caused bronchopneumonia, while in the cardiovascular system metastrongyles caused vasculitis in nearly half of the studies (45.45%, *n* = 5/11) that assessed these tissues. Metastrongyles were associated with otitis in 23.08% (*n* = 6/26) of the studies. Metastrongyle infection was considered a potential contributory to mortality in 44.78% (*n* = 90/201) of the studies while 10.45% (*n* = 21/201) of these studies considered metastrongyles the main cause of death. Metastrongyle species with a wider host range were more likely to induce pathogenic effects. Metastrongyles can cause significant tissue damage and mortality in marine mammals although virulent host–parasite relationships are dominated by a few metastrongyle species with wider host ranges.

## Graphical abstract

**Figure:**
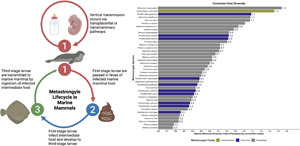

## Introduction

Metastrongyloidea (Nematoda: Strongylida), also known as lungworms or metastrongyles, are common parasites of the respiratory, cardiovascular and rarely the auditory system of mammals (Measures, 2001). The definitive hosts become infected through ingestion of larval stages within invertebrate intermediate hosts or free on pasture; therefore, metastrongyle infection can be quite common among free-foraging domestic and wild mammals (Measures, 2001; Regassa *et al*., 2010; Niebuhr *et al*., 2021). Like other helminth infections, the pathogenic effects of metastrongyloidiasis can be broad with impacts that range from subclinical infection to bronchopneumonia and death (Measures, 2001; Forbes, 2021). The reasons for these variable outcomes are not fully understood but site of infection (e.g. upper *vs* lower airways), burden and host immune status have been indicated as drivers of pathogenic outcomes (Forbes, 2021). However, it is unclear if certain host–parasite relationships are more detrimental for the host and if these more virulent associations obey to ecological factors that could illuminate conserved drivers of pathogenicity in these nematodes. Interestingly, the virulence of metastrongyles could be higher for animal species that require optimal respiratory and auditory function for fitness, such as marine mammals (Bergeron *et al*., 1997; Measures, 2001; Seibel *et al*., 2010). Marine mammals rely on cardiovascular and lung capacity for foraging dives and the otic apparatus of cetaceans is critical for communication and echolocation in the water column (Bergeron *et al*., 1997; Measures, 2001; Seibel *et al*., 2010). Therefore, metastrongyles parasitism of these host compartments could be associated with disease, stranding and death (Bergeron *et al*., 1997; Measures, 2001; Seibel *et al*., 2010), making this animal group an interesting model to test conserved correlates of virulence among metastrongyle species. Nevertheless, metastrongyles are one of the most diagnosed parasites in marine mammals, and in many circumstances are considered incidental findings of little to no pathologic significance (Geraci and St. Aubin, 1987). This conflicting evidence could be related to negative metastrongyle effects limited to specific host–parasite relationships, parasite traits, environmental conditions or the research methods employed to assess the health impacts. However, a systematic assessment of the factors that could modulate the health impacts of metastrongyles has not been reported.

**Figure 1.**
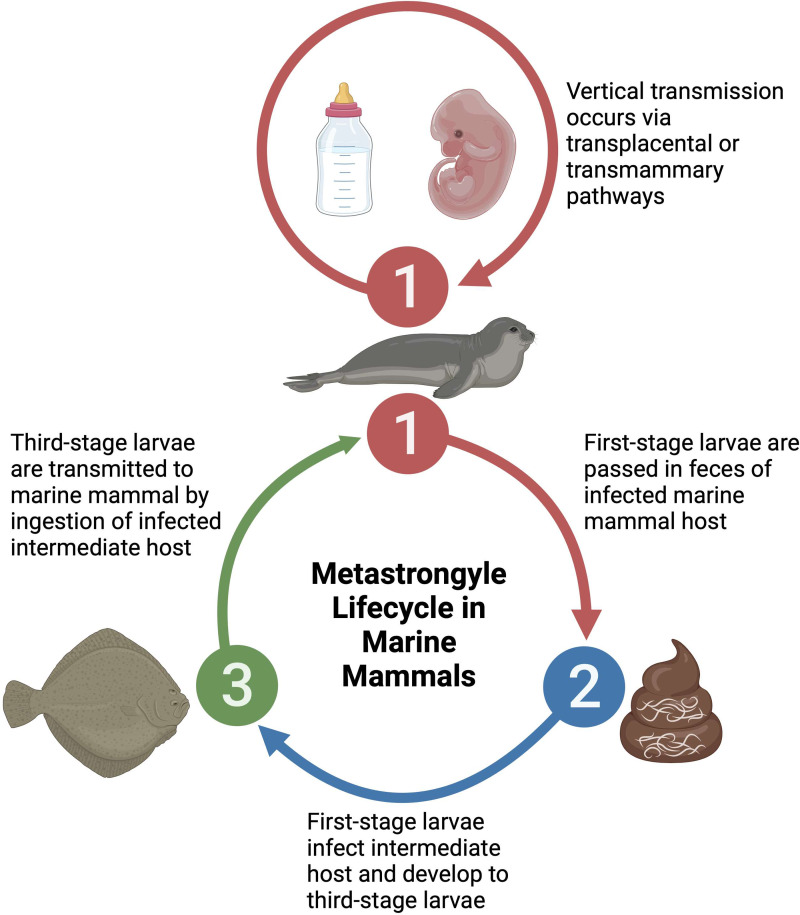
Generic lifecycle of metastrongyles in marine mammals. (1) Hosts become infected through consumption of intermediate hosts containing infective larvae. Alternatively, young animals can become infected through consumption of larvae in the mother's milk or *in utero* through transplacental infection. (2) Infected hosts release larvae or embryonated eggs in feces. (3) Larvae are consumed or infect intermediate hosts, usually a fish species. Created with BioRender.com; publication and licencing agreement number: RP25UI473J.

Three families of metastrongyles are prominently found in marine mammals, spanning 7 genera, with a considerable diversity of species within each genus. Despite this diversity, metastrongyles share similar life histories and host exploitation strategies in marine mammals. A generic metastrongyle lifecycle in marine mammals initiates with fecal shedding of first-stage larvae by an infected marine mammal host, resulting in the infection of an intermediate host such as a fish which feeds on fecal material (Dailey, 1970; Bergeron *et al*., 1997). Within the intermediate host, the first-stage larvae mature into third-stage larvae, which are infective to marine mammals upon ingesting the intermediate host (Dailey, 1970; Bergeron *et al*., 1997). Additionally, it is suspected that transplacental or transmammary vertical transmission of metastrongyles may occur in some species, serving as an alternative lifecycle pathway (Dailey *et al*., 1991; Balbuena *et al*., 1994; Fauquier *et al*., 2009; Reckendorf *et al*., 2018) (Fig. 1). Consumed metastrongyle larvae migrate from the gastrointestinal system to the respiratory or cardiovascular system where sexual maturity and reproduction occurs, releasing embryonated–larvated eggs or larvae (Taylor *et al*., 2015).

In the airways and respiratory sinuses or cardiovascular system, metastrongyles feed on epithelial–endothelial cells, host debris and occasionally blood (Dailey, 1970; Fauquier *et al*., 2009; Rhyan *et al*., 2018). These processes of migration through tissues, feeding and releasing eggs and larvae illicit variable immune responses in the host (Baylis and Daubney, 1925; Fauquier *et al*., 2009; Groch *et al*., 2020). This immune response is usually directed against parasite cuticle, larvae, eggs or parasite remains and usually consist in the attraction of macrophages, eosinophils and lymphocytes to the site of infection (Fauquier *et al*., 2009; Zafra *et al*., 2015; Groch *et al*., 2020; Seguel *et al*., 2020). The degree of this inflammatory response and its impact on health status can be quite variable ranging from minimal to severe bronchopneumonia (Fauquier *et al*., 2009; Groch *et al*., 2020; Seguel *et al*., 2020), being the drivers of the variable responses poorly understood.

There is conflicting evidence regarding the connection between metastrongyle infections and cetacean strandings (Arbelo *et al*., 2013). Currently, there is no consensus on whether inflammation in the otic apparatus, elicited by a presence of metastrongyle parasites, can impair hearing or echolocation to a significant enough degree to be considered a contributory factor in cetacean strandings (Geraci and St. Aubin, 1987; Wohlsein *et al*., 2019). Similarly, the presence and feeding behaviour of the nematodes in bronchi and lung parenchyma facilitates secondary bacterial infections with conflicting documented outcomes, ranging from little importance for lung physiology to fatal bronchopneumonia, especially when parasites are found in the lower respiratory system (Siebert *et al*., 2001; Lehnert *et al*., 2010; Ulrich *et al*., 2015, 2016; Reckendorf *et al*., 2021). This conflicting evidence could be related to several host and parasite traits, although across biological systems, parasite biomass, usually assessed in terms of burden, is a common driver of negative health outcomes (Pedersen and Fenton, 2015; Seguel and Gottdenker, 2017). Similarly, parasite and host sizes play an important role in assessing total parasite biomass and can sometimes serve as useful proxies of biomass to identify particularly detrimental host–parasite relationships across studies (Seguel and Gottdenker, 2017). Another potential factor impacting health outcomes across host–parasite systems is the capacity of certain helminths to parasitize multiple host species. Parasites with a wider host range can potentially become more virulent because decreases in host fitness due to infection can be compensated by infecting other susceptible species (Poulin, 2011; Schmid-Hempel, 2021). However, the role of these host and parasite traits on the health impact of metastrongyles is not well defined.

The objectives of this systematic review were to (i) summarize existing literature concerning relationships between marine mammals and metastrongyle parasites, (ii) assess the relative host and parasite diversity of infections and (iii) outline the pathology and health consequences that result from metastrongyle infections in marine mammals, with assessment of the potential drivers of these effects.

## Materials and methods

### Searching methods and inclusion criteria

A systematic literature review on metastrongyle parasites in marine mammals was conducted on 6 July 2021. The search was conducted using Google Scholar and PubMed, following the best practices outlined by Haddaway and Watson (2016) to conduct an aggregative systematic review in the field of parasitology and the recommendations outlined in the ‘Preferred Reporting Items for Systematic Reviews and Meta-Analyses’ (PRISMA) (Haddaway and Watson, 2016; Page *et al*., 2021).

The search terms used for the literature search on PubMed were as follows: ‘[(Marine) OR (Cetacean) OR (Delphinidae) OR (Phocidae) OR (Phocoenidae) OR (Otariidae) OR (Monodontidae) OR (Mustelidae) OR (Trichechidae) OR (Odobenidae) OR (Ursidae) OR (Odobenidae) OR (Balaenidae) OR (Neobalaenidae) OR (Eschrichtiidae) OR (Balaenopteridae) OR (Physeteridae) OR (Kogiidae) OR (Ziphiidae) OR (Platanistidae) OR (Iniidae) OR (Lipotidae) OR (Pontoporiidae) OR (Dugongidae) OR (Seal) OR (Porpoise) OR (Otter) OR (Dolphin) OR (Sea lion) OR (Whale) OR (Walrus) OR (Manatee)] AND [(Metastrongyle) OR (Metastrongylus) OR (Metastrongyloidea) OR (Metastrongylidae) OR (Parafilaroides) OR (Stenurus) OR (Halocercus) OR (Pseudaliidae) OR (Filaroididae) OR (Crenosomatidae) OR (Otostrongylus) OR (Skrjabingylus)]’.

The search terms used for the literature search on Google Scholar were reduced due to limitations in the advanced search software, notably a 256-character limit on searches. The search terms used for Google Scholar were as follows: ‘[(Metastrongyle) OR (Metastrongylus) OR (Metastrongyloidea) OR (Metastrongylidae) OR (Parafilaroides) OR (Stenurus) OR (Halocercus) OR (Pseudaliidae) OR (Filaroididae) OR (Crenosomatidae) OR (Otostrongylus) OR (Skrjabingylus)] AND [(Marine) OR (Cetacean)]’.

**Figure 2.**
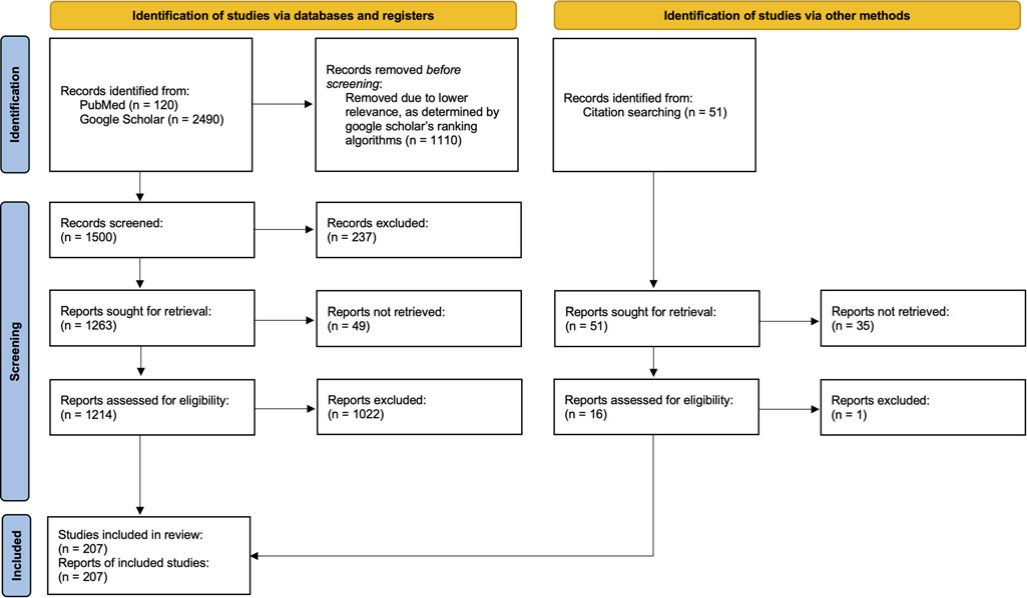
Flow diagram outlining the identification and screening process of manuscripts for inclusion in this study, in accordance with the PRISMA 2020 guidelines for systematic reviews.

The search on PubMed yielded 120 results and the search on Google Scholar yielded 2110 results, excluding citations. As Google Scholar can only display 1000 results per search, only the 1000 articles deemed most relevant by the software's ranking algorithms were manually reviewed for inclusion in the study, in addition to the 120 articles found *via* PubMed. To ensure all recently published literature was consulted, a secondary search on google scholar was conducted with the same search terms, filtering to only include articles published from 2017 and onward. This secondary search yielded 380 results, excluding citations, which were also manually reviewed for inclusion in the study. All conference archives (e.g. IAAAM), theses not published in peer-reviewed journals and posters were excluded from this study. The full texts for the articles isolated through literature search results were obtained and manually reviewed by the 2 authors, being included in the study if they met the following inclusion criteria: (i) the host species was any wild marine mammal, with captive wild animals only included if the transmission of parasites was stated to have occurred naturally, prior to their capture. (ii) The parasite species belonged to a genus within the metastrongyle superfamily, including studies that described the presence of parasitic eggs and larvae provided they could be positively identified to at least the genus level (studies that described parasites as ‘lungworms’ or ‘nematodes’ without specificity regarding genera were excluded). (iii) The article serves as the primary source describing a relationship between a marine mammal host and metastrongyle parasite. Duplicate articles, existing in both the Google Scholar and PubMed searches, were manually removed. All non-primary sources which were found through the initial search terms were screened, and citations were followed, leading to the inclusion of 15 additional articles which met the inclusion criteria. Later, the World Register of Marine Species (WoRMS, https://www.marinespecies.org), the Society for Marine Mammalogy (https://marinemammalscience.org) and the Natural History Museum Host-Parasite database (https://www.nhm.ac.uk/research-curation/scientific-resources/taxonomy-systematics/host-parasites/) were consulted to ensure all known unique relationships were reported in this study (WoRMS Editorial Board, 2022). A total of 35 articles could not be acquired in their full text form for inclusion in data analysis, but the relationships they report are known through prior citations in peer-reviewed journals or available abstracts. The articles which met the inclusion criteria were further evaluated by the 2 authors, with significant attributes documented on a master spreadsheet. Many included articles, including the articles not available in full-text form, report multiple different host–parasite relationships, thus independent rows were created on the master spreadsheet for each described host–parasite relationship (*n* = 481), allowing for relationship-specific attributes to be evaluated. The accepted common names and scientific names provided for host species in this study are taken from the Society for Marine Mammalogy Committee on Taxonomy (WoRMS Editorial Board, 2022). A flow diagram outlining the identification and screening process used in this review is included, which conforms with the ‘PRISMA 2020 Statement’ guidelines for systematic reviews (Fig. 2) (Page *et al*., 2021).

A total of 207 full-text publications met the inclusion criteria for this study, with 114 appearing only on Google Scholar, 6 appearing only on PubMed, 72 appearing on both Google Scholar and PubMed, and 15 coming from other sources. Data were collected on all included full-text publications, including whether they assessed pathogenic effect. An article was deemed to assess pathogenic effect if it described some biological impact associated with a presence of metastrongyle parasites in the host, or it explicitly stated no pathogenic effect was associated with the parasitism. Further data were collected on whether full-text publications which met the inclusion criteria mentioned strandings or mortality. For the collection of these data, an article was deemed to mention strandings if it contained the following terms (not including in the citations): ‘stranded’ or ‘stranding’ or ‘strandings’. For an article to have been deemed to mention mortality, it must have contained the following terms (not including in the citations): ‘mortality’ or ‘mortalities’. The inclusion terms for determining which articles mention stranding or mortality were isolated through word searching in adobe acrobat, with technologically non-conforming articles screened manually. Later, the studies mentioning mortality or stranding were assessed to ensure they discussed these effects in the context of the study's findings. The parasite collection methodology was also noted for each included full-text publication. Within the articles which met the inclusion criteria, the geographical location(s) were recorded for 437 documented relationships and the anatomical location(s) of the parasites were recorded in 371 documented relationships. We further calculated the geographical dispersion of the 102 unique relationships between marine mammals and metastrongyles.

For each documented relationship, data were also recorded on any reported pathogenic effects (*n* = 201). Pathogenic effects can be described as some biological impact on the host associated with the presence of metastrongyle parasites. In cases where it was explicitly stated that no pathogenic effect was associated with parasitism, this was recorded as well. Of the 101 articles that assessed pathogenic effect, there were a total of 201 relationships in which attributes describing a pathogenic effect or lack thereof were documented.

Between the 207 full-text publications which met our inclusion criteria, and the additional 35 publications we could not retrieve in full-text form, a total of 481 host–parasite relationships are documented. For each of these relationships, data were collected on their respective species taxonomy. Next, the frequency at which each metastrongyle parasite species appears in the literature, in the context of relationships with marine mammal hosts was recorded. For the purposes of frequency calculations, parasites described only to the genus level were excluded. The frequency at which marine mammal hosts appear in the literature, in the context of relationships with metastrongyle parasites was recorded in the same manner. For frequency calculations, hosts described only to the genus level were excluded. For the purposes of all diversity calculations, only relationships where both the host and parasite were described to the species level were considered. Finally, host diversity was calculated as a measure of the diversity of host species a metastrongyle species can infect, and parasite diversity was calculated as the diversity of metastrongyle species a marine mammal species can host.

To provide a metric by which the size of a host could be used as a predictor of pathogenic effects, host body mass was calculated by dividing the median weight in kilograms by the median length in centimetres for each host species. Median host measurements were compiled as the median of both genders from a variety of sources based on availability in the following order of priority: (1) WoRMS (https://www.marinespecies.org); (2) Society for Marine Mammalogy (https://marinemammalscience.org/science-and-publications/species-information/facts/); (3) National Oceanic and Atmospheric Administration (NOAA, https://www.fisheries.noaa.gov/species-directory); (4) Animal Diversity Web (ADW, https://animaldiversity.org). In cases where data on host size were not available through those 4 sources, the median measurements of hosts documented during gross necropsy in articles which met the inclusion criteria of this study were used.

Finally, we produced an up-to-date checklist of all known relationships concerning metastrongyles in marine mammals, which is included in Supplementary Table 1, complete with information on anatomical infection sites documented for each relationship. The relationships documented are found within the 207 articles included in this study or within the 35 articles that could not be acquired in their full-text form for inclusion in data analysis, provided the relationships they report are known through prior citations in peer-reviewed journals.

### Data management and analyses

When assessing the relative host and parasite diversities, corrections were made for literature and intra-study biases which arose from non-uniform sampling efforts. We used a modified approach, based on previously published methods (Nunn *et al*., 2003; Ezenwa *et al*., 2006; Seguel and Gottdenker, 2017), to create a bias penalization index for each host and parasite species. To do this, we searched scientific names of each host and parasite species individually on Google Scholar and the number of hits was recorded. The number of hits for each species was then multiplied by the number of studies that both met our inclusion criteria and contained mentions of a relationship concerning that respective species. The natural logarithm of these products was then taken, and the result constitutes our penalization index for each species study bias (correction-index). The resulting corrected diversity indexes were plotted using frequency plots constructed in the R package ‘ggplo2’.

We explored the predictors of pathogenic effects among different host parasite interactions using binomial generalized linear mixed-effects models (GLMMs) with the restricted maximum likelihood fitted using the R package ‘glmmTMB’ (Brooks *et al*., 2017). Although this method can estimate positively biased odds ratios for binary outcomes in metadata, these are usually more problematic for smaller sample sizes and alternative methods for the analysis of binary metadata present similar issues (Stijnen *et al*., 2010; Bakbergenuly and Kulinskaya, 2018). We used corrected host diversity, corrected parasite diversity and host body size as predictors for the presence or absence of pathogenic effects. Study ID was added as a random effect to account for studies documenting multiple host–parasite relationships with their respective outcomes. For each documented host–parasite relationship, with both the host and parasite specified to the species level and for which pathogenic effect was assessed (*n* = 165), binomial GLMMs for the presence or absence of pathogenic effect were fit. A second model was fit to exclusively assess the presence or absence of respiratory-specific effect (bronchopneumonia or bronchitis). For the respiratory-specific effect model, each documented host–parasite relationship was included if the host and parasite were specified to the species level, pathogenic effect was assessed and the presence of the parasite was documented in either the upper or lower respiratory system (*n* = 134). We attempted to fit cardiovascular-specific effect (DIC, thrombosis, vasculitis, haemorrhage, endocarditis) (*n* = 11) and otic apparatus-specific effect (sinusitis, otitis, otic metaplasia) (*n* = 24) models using the same method as for respiratory-specific effect but were unable because the small sample size affected model convergence. In both the full and respiratory-specific models, we included interaction effects between the corrected host diversity, corrected parasite diversity and body size predictor terms to account for individual permissiveness of host species to parasite species as well as individual pervasiveness of parasite species to host species.

### Discrepancies in the literature

Hermosilla *et al*. (2015) report *Stenurus sp*. in sei whale (*Balaenoptera borealis*), blue whale (*Balaenoptera musculus*) and fin whale (*Balaenoptera physalus*) as non-primary findings; however, we could not identify a primary source of these relationships (Hermosilla *et al*., 2015). Lehnert *et al*. (2019) report *Pharurus alatus* as a parasite of short-beaked common dolphin (*Delphinus delphis*) citing Tomo *et al*. (2010) (Lehnert *et al*., 2019). Upon reviewing Tomo *et al*. (2010), it does not appear to include this relationship (Tomo *et al*., 2010). The WoRMS documents *Halocercus taurica* in short-beaked common dolphin (*D. delphis*) and *Halocercus hyperoodoni* in northern bottlenose whale (*Hyperoodon ampullatus*); however, primary sources could not be identified for either of these relationships (WoRMS Editorial Board, 2022). Measures (2001) documents *Halocercus pingi* in Dall's porpoise (*Phocoenoides dalli*) as a non-primary finding; however, we could not find a primary source for this relationship (Measures, 2001). Three (*n* = 3) additional publications document relationships that were not specified to the genus level (Giorda *et al*., 2017; Reckendorf *et al*., 2021; van Wijngaarden *et al*., 2021).

## Results

At least 40 species of metastrongyles have been described in 66 marine mammal host species. Most metastrongyle species parasitize multiple host species, with a total of 213 unique host–metastrongyle relationships recorded (Supplementary Table 1).

### Characterization of studies

**Figure 3.**
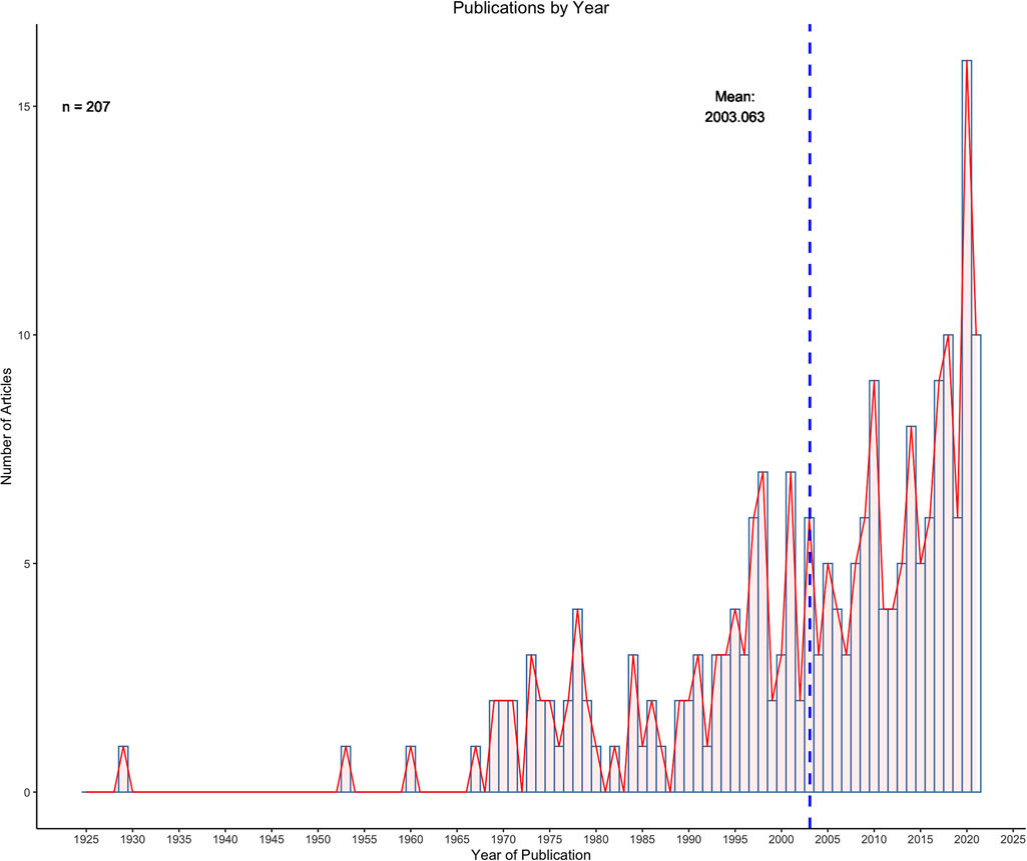
Distribution of publication year for articles which met the inclusion criteria of this study. The mean year of publication was 2003.

**Figure 4.**
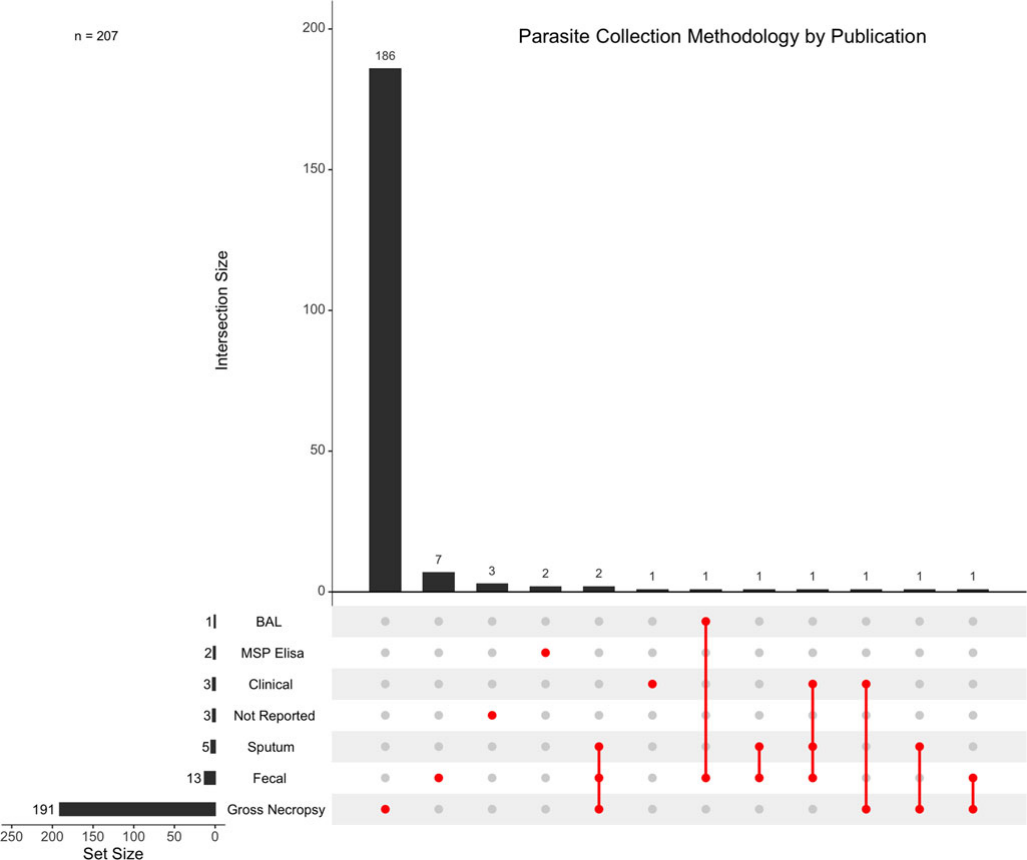
Parasite collection methodologies documented, with intersections between the methodologies shown to represent studies that utilize more than 1 methodology. Articles that did not report a collection methodology are designated as ‘Not Reported’.

The mean publication year of the 207 articles which met the inclusion criteria was 2003, with the distribution of publication years skewed towards the most recent decade (Fig. 3). For each of the articles which met the inclusion criteria, 48.88% (*n* = 101/207) assessed pathogenic effect. Additionally, 78.74% (*n* = 163/207) of studies mentioned stranding and 43.48% (*n* = 90/207) of articles mentioned mortality as potentially related to lungworm infection. Gross necropsy is the predominant parasite collection method for metastrongyles in marine mammals with 92.27% (*n* = 191/207) of the studies using this as the only method of collection. The next most common method of collection was fecal sampling in 6.28% (*n* = 13/207) of the studies (Fig. 4).

**Figure 5.**
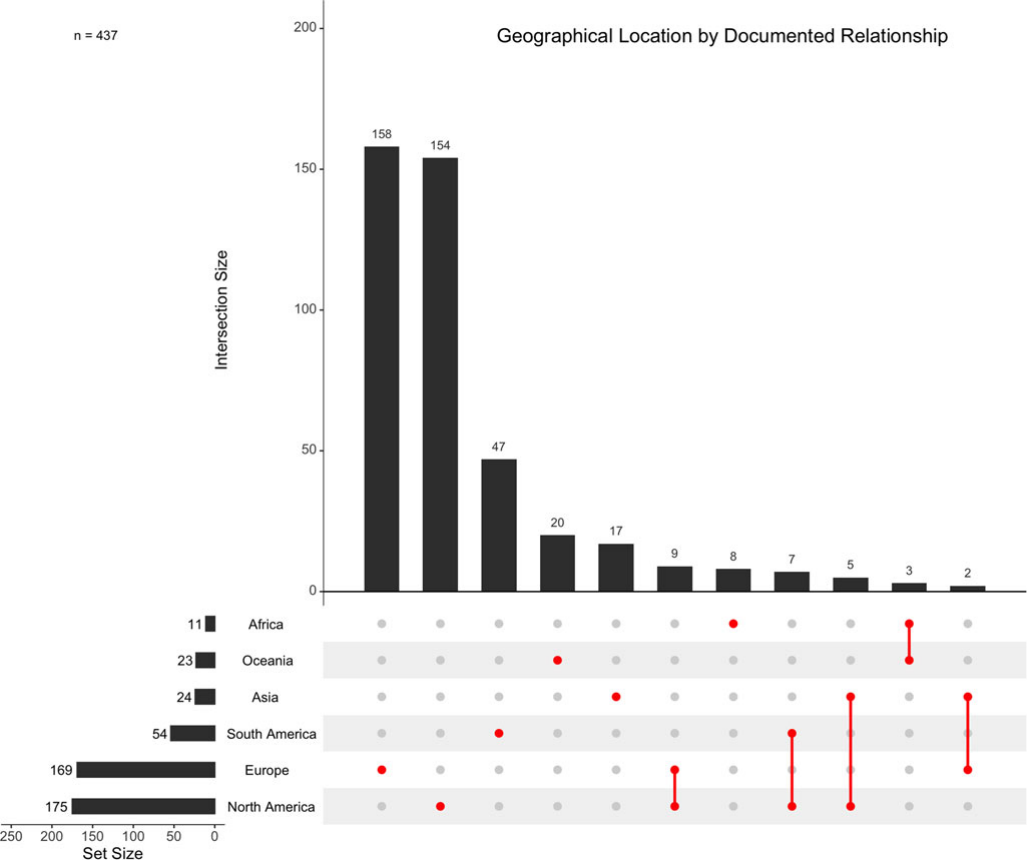
Geographical location(s), denoted by continent, of documented relationships between metastrongyles and marine mammals. Documented relationships include duplicates of unique relationships published independently. Intersections are shown for studies which report individual host species to parasite species relationships occurring in multiple locations.

**Figure 6.**
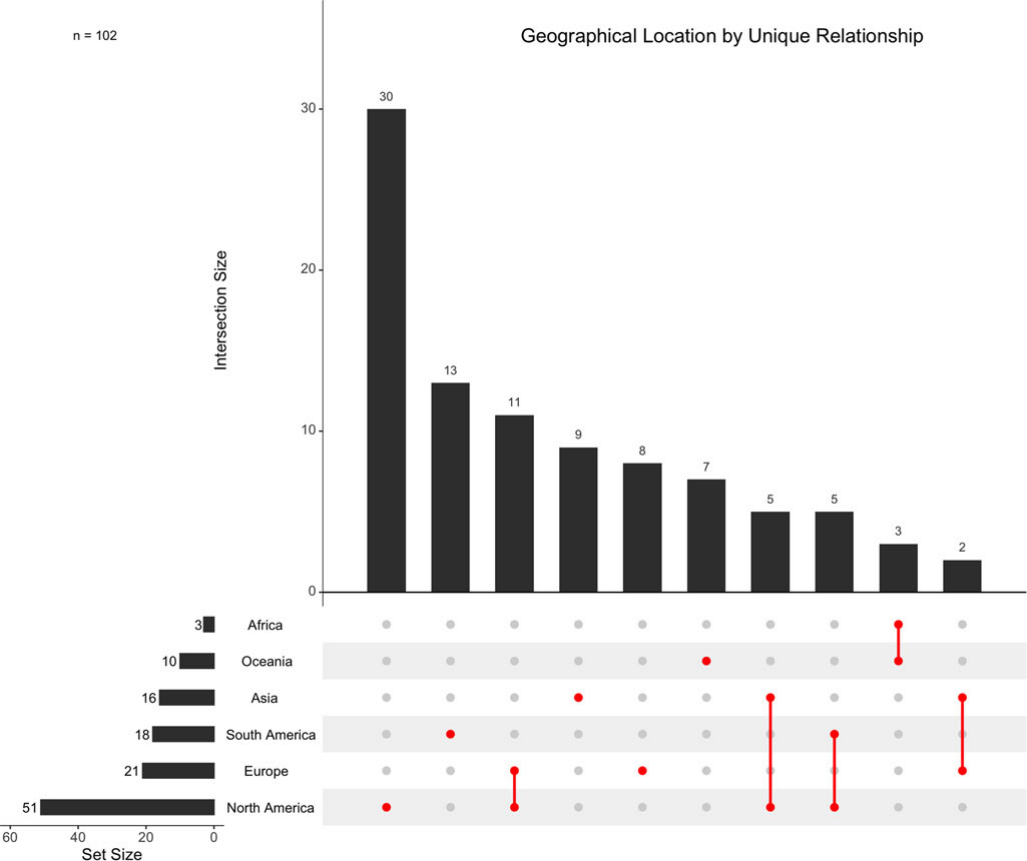
Geographical location(s), denoted by continent, of all unique relationships between metastrongyles and marine mammals. Intersections are shown for studies which report individual host species to parasite species relationships occurring in multiple locations.

A total of 175 host–metastrongyle relationships were reported in North America (including the commonly referenced Central America region), and 169 relationships were reported in Europe. North America and Europe are the most common locations reported for metastrongyle infections in marine mammals, followed by 54 relationships in South America, 24 in Asia, 23 in Oceania and 11 in Africa (Fig. 5). Exactly half (50.00%, *n* = 51/102) of all unique relationships are reported in North America, with 29.41% (*n* = 30/102) of these relationships being reported exclusively in North America (Fig. 6).

### Frequencies and diversities of Metastrongyloidea infections in marine mammals

Pseudaliidae was the most reported metastrongyle family in marine mammals (*n* = 331), followed by Filaroididae (*n* = 90) and Crenosomatidae (*n* = 58). At the host family level, Delphinidae (*n* = 159), Phocoenidae (*n* = 150) and Phocidae (*n* = 107) are most frequently parasitized by metastrongyles. Further, metastrongyles from the Pseudaliidae family are reported in hosts from the Delphinidae (*n* = 158), Phocoenidae (*n* = 148), Monodontidae (*n* = 15), Ziphiidae (*n* = 3), Iniidae (*n* = 2), Balaenopteridae (*n* = 1), Kogiidae (*n* = 1) and Physeteridae (*n* = 1) families. Metastrongyles from the Crenosomatidae family are reported to parasitize hosts from the Phocidae (*n* = 52), Otariidae (*n* = 5) and Phocoenidae (*n* = 1) families. Metastrongyles from the Filaroididae family are reported to parasitize hosts from the Phocidae (*n* = 54), Otariidae (*n* = 35) and Delphinidae (*n* = 1) families.

*Otostrongylus circumlitus* (*n* = 52) is the most reported metastrongyle species followed by *Stenurus minor* (*n* = 42) and *Pseudalius inflexus* (*n* = 31) (Supplementary Fig. 1). The harbour porpoise (*Phocoena phocoena*) (*n* = 118) is the most reported host, followed by harbour seal (*Phoca vitulina*) (*n* = 41) and bottlenose dolphin (*Tursiops truncatus*) (*n* = 23) (Supplementary Fig. 2).

**Figure 7.**
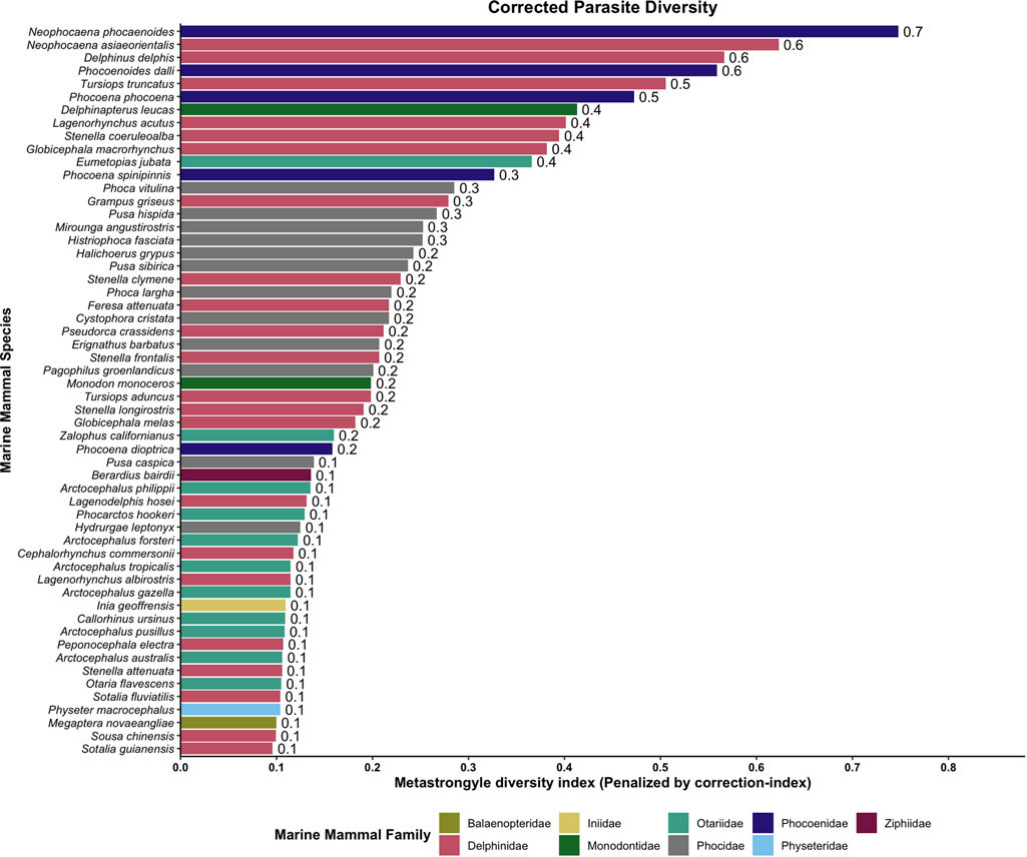
Parasite diversity for metastrongyles in marine mammals, corrected for publication bias (correction index).

**Figure 8.**
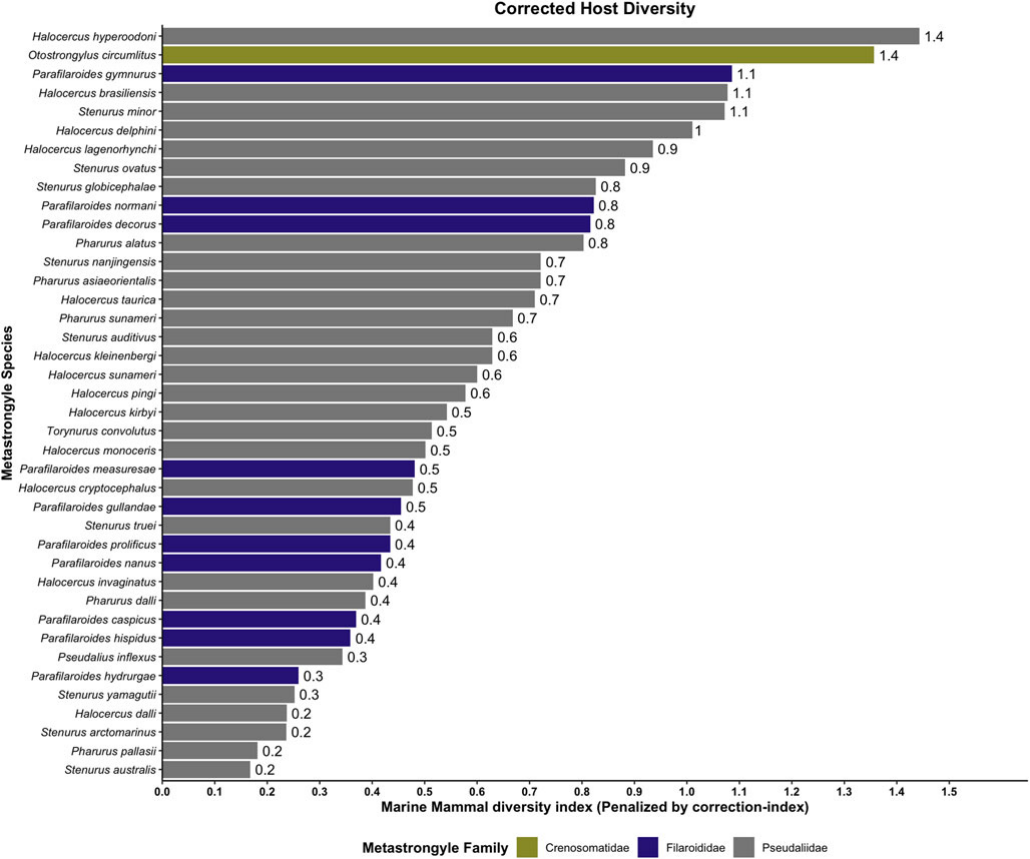
Host diversity for metastrongyles in marine mammals, corrected for publication bias (correction index).

Before penalizing for literature and intra-study biases, the host species harbouring the largest number of metastrongyle species (parasite diversity) were: indo-pacific finless porpoise (*Neophocaena phocaenoides*) (*n* = 8), short-beaked common dolphin (*D. delphis*) (*n* = 7), harbour porpoise (*P. phocoena*) (*n* = 7) and bottlenose dolphin (*T. truncatus*) (*n* = 7) (Supplementary Fig. 3). After correction for publication bias, indo-pacific finless porpoise (*N. phocaenoides*) remained the most permissive host (Fig. 7). Before penalization, the metastrongyle species with the broader host diversity were: *O. circumlitus* (*n* = 13), *S. mino*r (*n* = 10), *Parafilaroides gymnurus* (*n* = 9), *Halocercus brasiliensis* (*n* = 8) and *Halocercus delphini* (*n* = 8) (Supplementary Fig. 4). After correction for publication bias, *Halocercus hyperdooni* emerged as the most generalist lungworm species, followed closely by *O. circumlitus* and *P. gymnurus*. In contrast, *Halocercus dalli*, *Stenurus arctomarinus*, *Pharurus pallasii* and *Stenurus australis* appeared to be highly host specific (Fig. 8).

### The impact of metastrongyle infections on marine mammals

**Figure 9.**
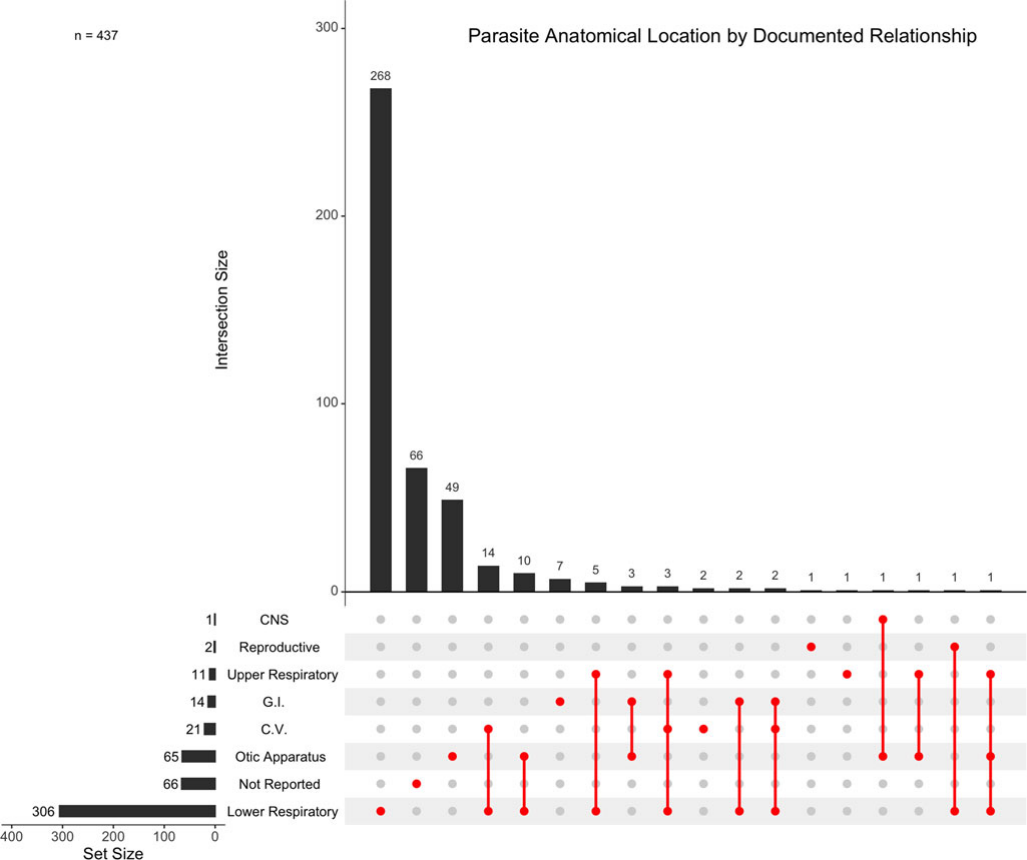
Anatomical location(s) of documented relationships between metastrongyles and marine mammals. Intersections are shown for studies which report individual host species to parasite species relationships occurring in multiple locations. Relationships that did not report an anatomical location are designated as ‘Not Reported’. CNS, central nervous system; GI, gastrointestinal tract; CV, cardiovascular system.

Of the 481 host–parasite relationships documented, 371 (77.13%) reported an anatomical location. Lungworms were found in the lower respiratory system in 82.48% (*n* = 306/371) of cases, while parasites were found in the otic apparatus in 17.52% (*n* = 65/371) of cases. In 11.59% (*n* = 43/371) of cases, a single species of parasite parasitized an individual host in multiple anatomical locations (Fig. 9).

**Figure 10.**
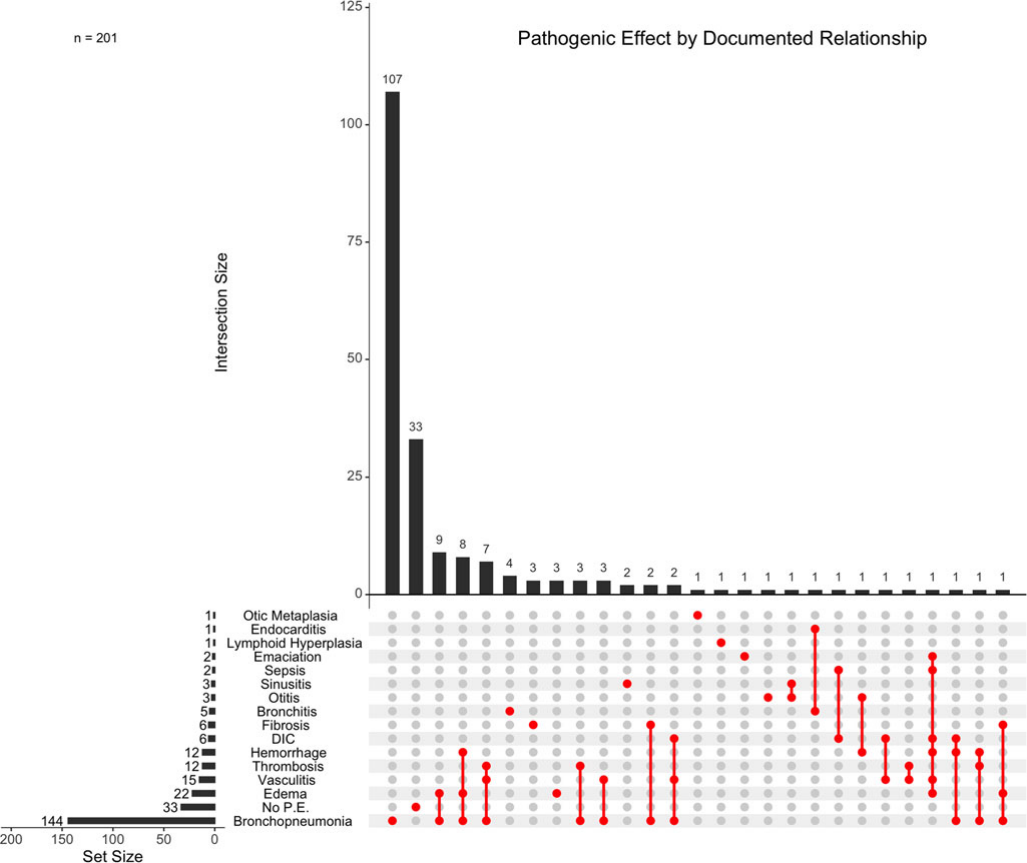
Pathogenic effect(s) of documented relationships between metastrongyles and marine mammals. Intersections are shown for studies which report individual host species to parasite species relationships causing multiple pathogenic effects. Relationships were designated as ‘No. P.E.’ when pathogenic effect was assessed and was determined to be negligible. DIC, disseminated intravascular coagulation.

Out of the 201 unique host–parasite relationships assessed, 44.77% (*n* = 90/201) mentioned mortality as ‘possible’ or ‘potential’ contributory cause of death and only 10.44% (*n* = 21/201) studies considered metastrongyle infection as the primary cause of death. Of the 201 host–parasite relationships for which pathogenic effect was assessed, bronchopneumonia was described in 71.64% (*n* = 144/201) of the relationships, indicating a high prevalence of bronchopneumonia in hosts infected with metastrongyles (Fig. 10). Other reported pathogenic effects included oedema (10.95%, *n* = 22/201), vasculitis (7.46%, *n* = 15/201), thrombosis (5.97%, *n* = 12/201) and haemorrhage (5.97%, *n* = 12/201). Lungworm parasitism resulted in no discernible pathogenic effect on the host in 16.42% (*n* = 33/201) of the relationships.

Pathogenic effect was assessed for 166 host–parasite relationships in the respiratory system, with respiratory-specific pathology reported in 80.12% (*n* = 133/166) of these cases. In contrast, 26 host–parasite relationships assessed pathogenic effect in the otic apparatus, with otic-specific lesions reported in only 23.08% (*n* = 6/26) of these relationships.

In the binomial GLMM that assessed pathogenic effect, host body size, corrected host diversity and corrected parasite diversity were all significant predictors of the presence of metastrongyle pathogenic effect in marine mammals, with a significant effect of the interaction between host and parasite diversity indexes (Table 1). For respiratory-specific effects, corrected parasite diversity and corrected host diversity main effects and their interaction were significant predictors of metastrongyle respiratory-specific effect in marine mammals, but host body size was not a significant predictor of respiratory-specific effect (Table 2).

**Table 1.** Binomial generalized linear mixed-effects model (GLMM) for presence of pathogenic effect

Predictor	Estimate	S.E.	*Z*	*P*
(Intercept)	−189.93	65.03	−2.921	0.00349
Corrected parasite diversity	523.62	165.18	3.170	0.00152
Corrected host diversity	232.05	88.44	2.624	0.00869
Host body size	166.90	83.04	2.010	0.04444
Corrected parasite diversity: corrected host diversity	−625.90	250.44	−2.499	0.01245
Corrected parasite diversity: host body size	−435.38	220.87	−1.971	0.04870
Corrected host diversity: host body size	−189.10	131.22	−1.441	0.14953
Corrected parasite diversity: corrected host diversity: host body size	581.44	438.86	1.325	0.18521

Presented are predictors estimates, standard error (S.E.), statistic (*Z*) and *P* values. The model used study ID as a random effect (total observations *n* = 165, study ID *n* = 89).

**Table 2.** Binomial generalized linear mixed-effects model (GLMM) for respiratory pathogenic effects

Predictor	Estimate	S.E.	*Z*	*P*
(Intercept)	0.09403	12.65860	0.007	0.99407
Corrected parasite diversity	88.93332	41.43432	2.146	0.03184
Corrected host diversity	74.10991	26.73963	2.772	0.00558
Host body size	−18.39291	13.61594	−1.351	0.17675
Corrected parasite diversity: corrected host diversity	−244.76967	87.16180	−2.808	0.00498
Corrected parasite diversity: host body size	8.03858	38.70274	0.208	0.83546
Corrected host diversity: host body size	−29.25975	24.54618	−1.192	0.23325
Corrected parasite diversity: corrected host diversity: host body size	154.98741	93.04556	1.666	0.09577

Presented are predictor estimates, standard error (S.E.), statistic (*Z*) and *P* values. The model used study ID as a random effect (total observations *n* = 134, study ID *n* = 76).

## Discussion and conclusions

Metastrongyles are common helminth parasites of the respiratory and cardiovascular system of mammals. Given the importance of these body systems for the survival of diving animals, it was expected that the health impacts of metastrongyles would be particularly significant in marine mammals. Our findings confirm that pathogenic effects, mainly bronchopneumonia, are common in marine mammals infected with metastrongyles, although association of metastrongyle parasites and mortality is usually not definitive. Importantly, our review suggests that pathogenic host–parasite relationships are associated to hosts that can harbour a wider range of metastrongyle species (parasite diversity). From the parasite perspective, metastrongyle species with a broader host diversity tend to be more pathogenic. These results emphasize the importance of host and parasite diversity with regards to the virulence of metastrongyles in marine ecosystems.

Most parasitology studies in marine mammals have been conducted through the opportunistic collection and examination of stranded carcasses. This probably explains in part why in most cases metastrongyle infections are suggested or considered as a contributory cause of death but rarely confirmed as the primary cause of mortality. Determining cause of death in stranded marine mammals is challenging, amid the rapid rate of postmortem changes in these species and little background information for most cases (Arbelo *et al*., 2013; Alvarado-Rybak *et al*., 2020). Additionally, access to carcasses is sometimes difficult (e.g. tides), limiting proper dissection techniques and tissue sampling. As with other host–parasite systems, the use of longitudinal assessments and experimental manipulation of infection could give new perspectives on the impact of metastrongyles in marine mammal health. Recently, the sampling of living species through the collection of feces and body fluids has been implemented in cetaceans (Williams *et al*., 2020). Further, the development of molecular techniques such as major sperm protein enzyme-linked immunosorbent assays to test for serum antibodies to metastrongyles aid in illuminating metastrongyle seroprevalence (Ulrich *et al*., 2015, 2016). The continued use of approaches other than opportunistic necropsies may substantially contribute to the understanding of the drivers and consequences of metastrongyle infection in marine mammals.

As with many wildlife parasites (Haddaway and Watson, 2016; Seguel and Gottdenker, 2017), most studies on metastrongyles in marine mammals are conducted in North America and Europe, leading to overrepresentation of North American and European marine mammal and metastrongyle species in the literature. Therefore, cosmopolitan coastal marine mammals in these regions such as the harbour porpoise and the harbour seal along with their parasites were among the most assessed species. Despite our intended penalization for these study biases, our results should be interpreted with caution since more studies from different regions of the world and in cryptic and/or pelagic marine mammal species could influence inferences on the role of host and parasite factors driving pathogenic effects. For instance, polychlorinated biphenyls (PCBs) and other persistent organic pollutants (POPs) are found in higher concentrations in marine mammals in more industrialized regions of North America and Europe (Fair *et al*., 2010; Durante *et al*., 2016; Jepson *et al*., 2016; Seguel *et al*., 2020). These immunosuppressive pollutants are thought to increase susceptibility to metastrongyles, facilitating their pathogenic effects (Jepson *et al*., 2016). Therefore, more studies from regions of the world with lower POP concentrations or where new or underrepresented host–parasite relationships occur could modify our understanding of the health impacts of metastrongyles in marine mammals.

Despite including only wild marine mammals in this review, it is impossible to determine in many cases if the host had previous exposure to semi-natural or captive environments such as nature sanctuaries and rehabilitation centres where infection could have occurred through contaminated intermediate hosts. Additionally, due to the highly migratory nature of many marine mammal species, the location at which the specimen was obtained may not reflect the geographical regions where infection occurred. Therefore, our compiled data on species distribution indicate parasite identification but it is not necessarily a good indicator of the phylogeography of metastrongyle infection range.

One challenge with discovering new or underrepresented host–parasite relationships is ensuring accurate metastrongyle identification and species delineation, particularly regarding more elusive species that have not undergone molecular characterization. As additional species are discovered, delineation *via* morphological features alone will become increasingly challenging. As such, molecular identification methods such as nucleotide sequencing have become increasingly necessary to ensure accurate species identification (Elson-Riggins *et al*., 2001; Gabel *et al*., 2021). The use of molecular identification methods is particularly beneficial in the case of metastrongyle hybridizations, which may arise due to the overlapping geographical ranges of species. The National Center for Biotechnology Information's ‘GenBank’ database currently includes rDNA markers for some of the more commonly researched metastrongyle species, including *O. circumlitus* and *S. minor* (Elson-Riggins *et al*., 2001; Gabel *et al*., 2021). However, it is important that as molecular inspection is performed on new or more elusive metastrongyle species, the determined nucleotide sequences are uploaded to ‘GenBank’, broadening the database's scope for metastrongyle species delineation.

As in domestic and land mammals, bronchopneumonia is the most common pathogenic outcome of lungworm infection in marine mammals. The presence of lungworms in airways or pulmonary parenchyma causes tissue damage, eliciting an inflammatory response that also facilitates colonization by bacteria (Measures, 2001). Therefore, in most of the assessed studies, verminous bronchopneumonia was usually complicated by secondary bacterial infection. The impact of metastrongyles in other tissues such as blood vessels, heart or otic apparatus is probably more direct, since in these tissues bacteria colonization secondary to helminth infection is rare. In the cardiovascular system, the impact of lungworm infection can be serious with vasculitis, localized or systemic thrombosis and coagulopathy (Gulland *et al*., 1997; Seguel *et al*., 2018; Barnett *et al*., 2019). In these cases, there is clear link between metastrongyle infection and mortality. Interestingly, invasion of the cardiovascular system is usually associated to metastrongyle species that are not the most common definitive host for that parasite species, highlighting the role of host specificity in the most pathogenic interactions between metastrongyles and marine mammals.

Parasite and host diversity increase the chances for a pathogenic outcome in metastrongyle–marine mammal relationships. This finding is supported by general hypotheses on parasite virulence that suggests that a parasite species with a greater diversity of potential hosts can evolve to become more pathogenic, as the sustainability of the parasite population is less dependent on the survival of individual hosts (Poulin, 2011; Schmid-Hempel, 2021). Similarly, a host species that harbours a greater diversity of parasites may see a greater propensity for pathogenic outcomes from individual parasite species due to an increase in the stress on the immune and respiratory systems resulting from co-infections (Seguel *et al*., 2023). Our results also suggest that larger hosts are more likely to sustain pathogenic outcomes because of metastrongyle parasitism. Since marine metastrongyles are transmitted through intermediate hosts that represent food items for marine mammals (Dailey, 1970), this result could indicate that increased consumption of food leads to greater larvae intake and subsequently a higher parasite burden. Similarly, a larger host body size could correlate with access to different prey items, contributing to a greater diversity of metastrongyle infections.

A comprehensive understanding of the drivers of the negative physiological effects associated with metastrongyle parasitism in marine mammal hosts is challenged by a presumably strong bias towards reporting only positive findings. The available literature clearly elucidates a multitude of host–parasite relationships in which metastrongyles produce a definable pathological effect; howbeit, it is inadequate in clarifying why these effects are observed in some species or populations and not in others. However, general host and parasite traits associated with pathogenic outcomes are like those of other helminth and microbial parasites, including host permissiveness of infection by multiple species and parasite capacity to infect multiple host species. In this context, the rapidly changing marine environment with generalized loss of biomass could favour parasites and hosts with a diversified trophic network, potentially selecting for more pathogenic relationships between metastrongyles and their marine mammal hosts.

## Supporting information

Fischbach and Seguel supplementary material 1 Fischbach and Seguel supplementary material

Fischbach and Seguel supplementary material 2 Fischbach and Seguel supplementary material

Fischbach and Seguel supplementary material 3 Fischbach and Seguel supplementary material

## Data Availability

The data that supports the findings of this study is available as supplementary materials.

## Supplementary material

The supplementary material for this article can be found at https://doi.org/10.1017/S0031182023001014.
