# Supplementary material for: A systematic review of the diversity and virulence correlates of metastrongyle lungworms in marine mammals
Source: Parasitology. 2023 Oct 20;150(13):1178–91. doi: 10.1017/S0031182023001014 (PMC10801380; doi:10.1017/S0031182023001014)
Supplement: Fischbach and Seguel supplementary material 2 — Fischbach and Seguel supplementary material [file S0031182023001014sup002.docx]

**Supplementary Material**

Title: A systematic review of the diversity and virulence correlates of metastrongyle lungworms in marine mammals

Authors: Fischbach, J.R. & Seguel, M.

Supplementary Figures:


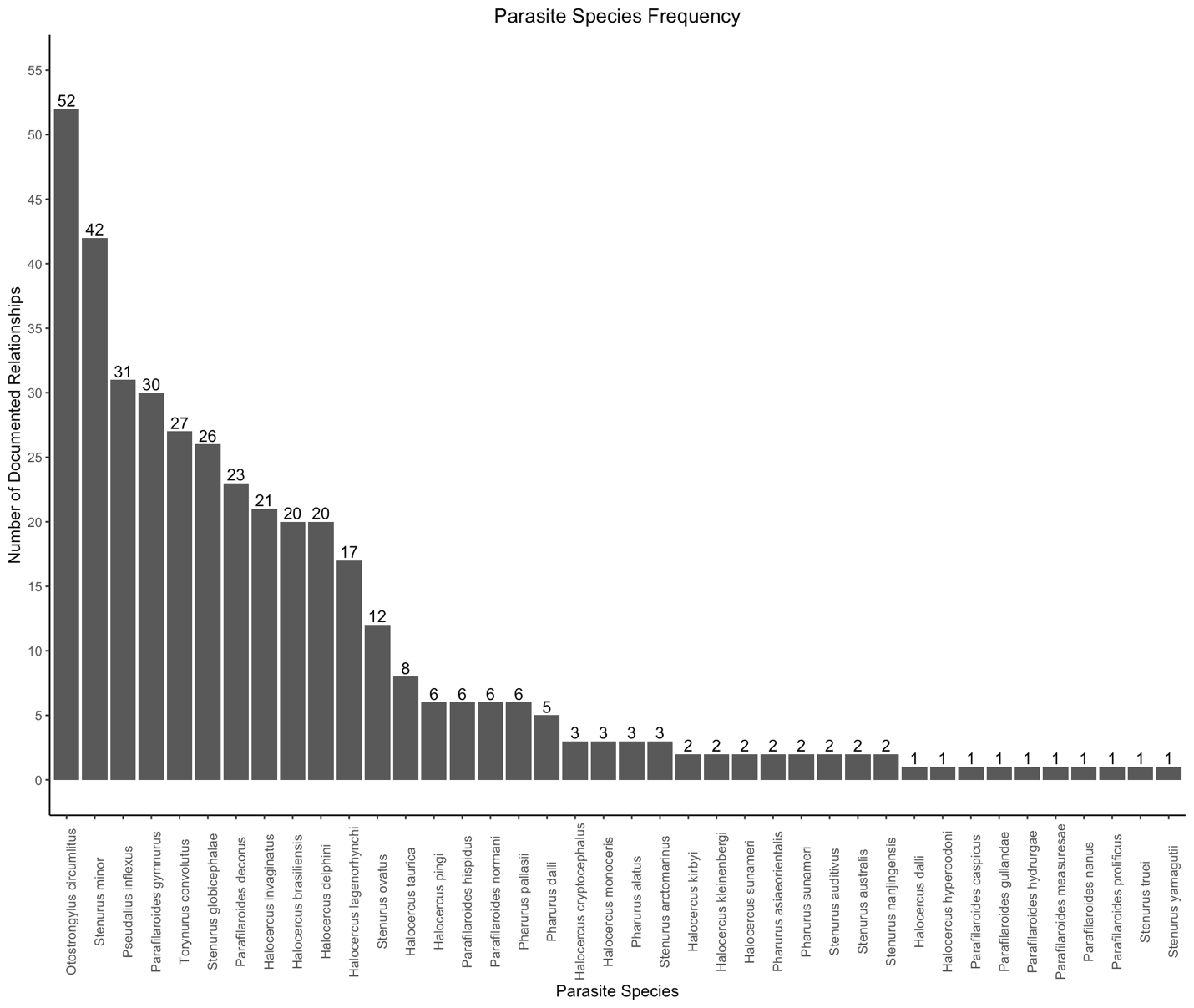


Supplementary Figure 1: Frequency of metastrongyle species documented.


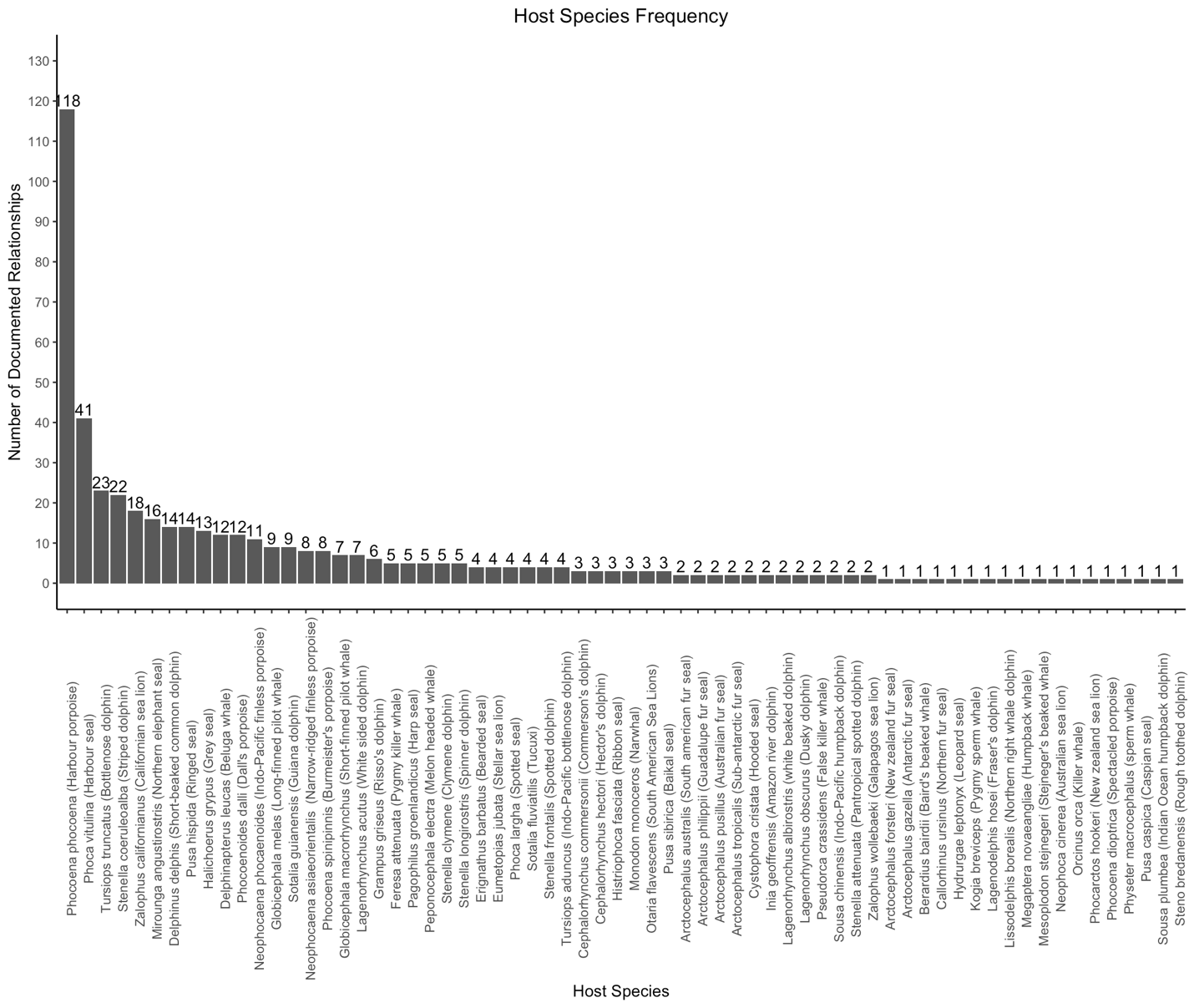


Supplementary Figure 2: Frequency of marine mammal host species documented.


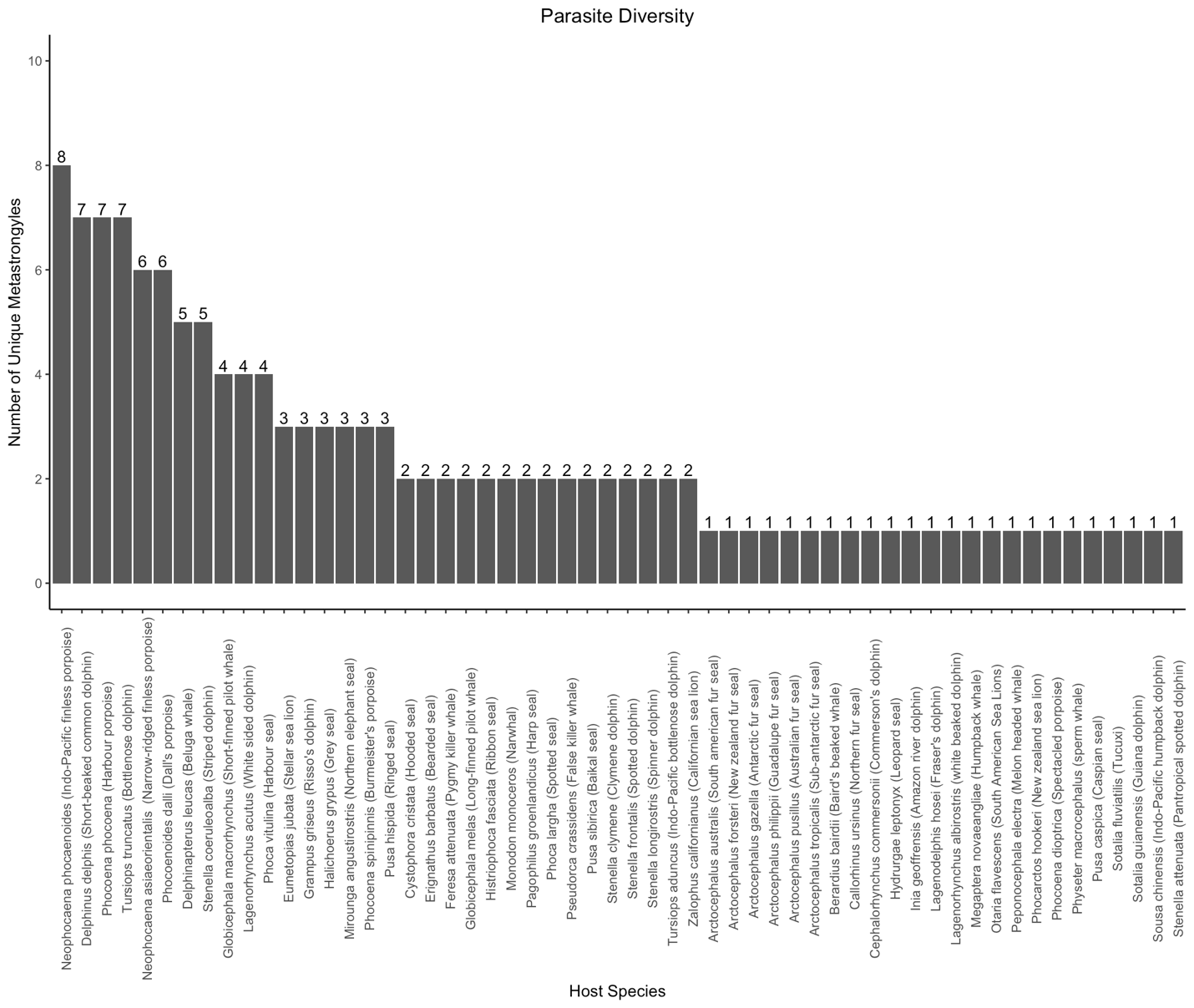


Supplementary Figure 3: Parasite diversity for metastrongyles in marine mammals (number of unique metastrongyle species hosted by each marine mammal).


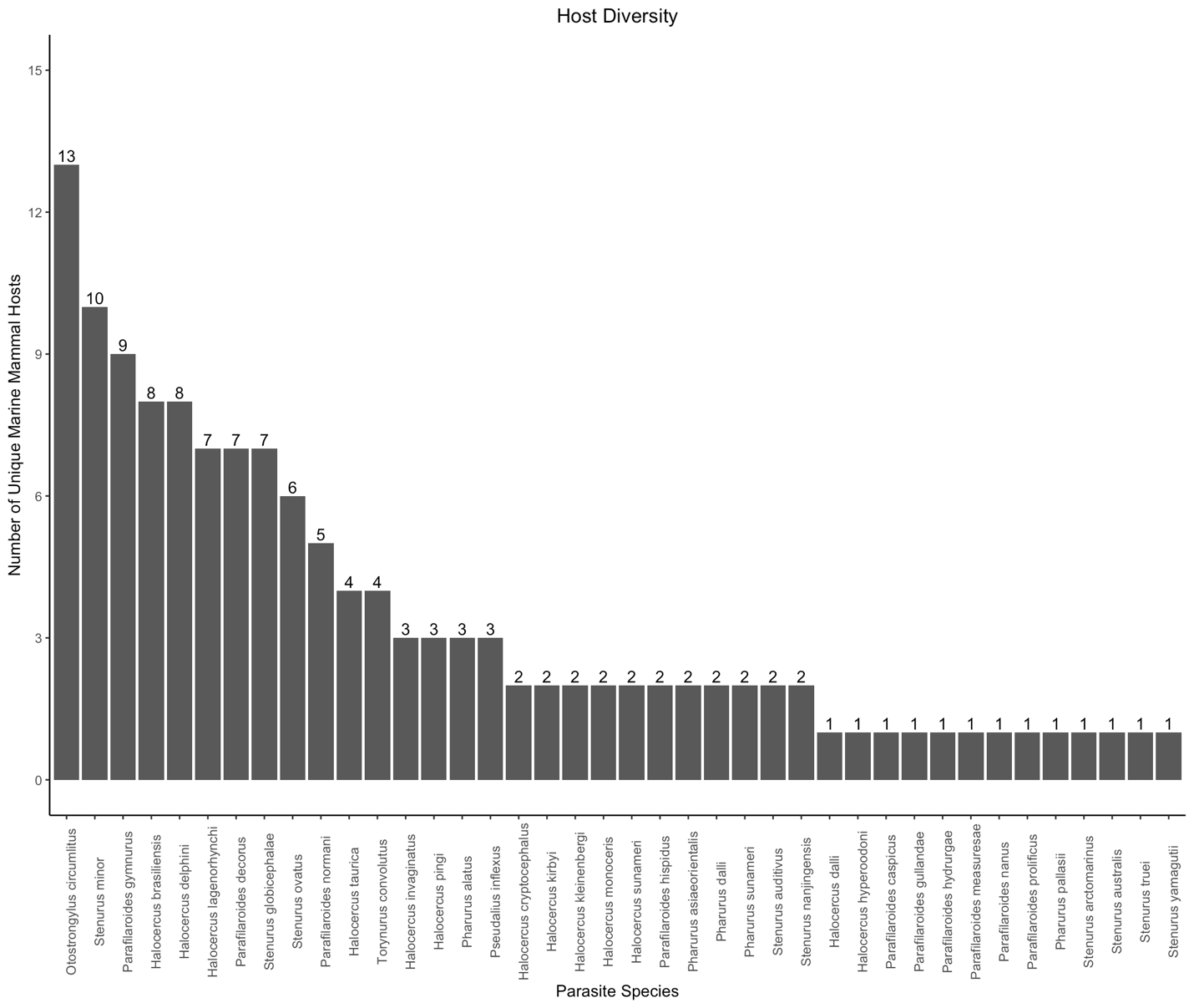


Supplementary Figure 4: Host diversity for metastrongyles in marine mammals (number of host species parasitized by each metastrongyle).
