# Supplementary material for: A systematic review of the diversity and virulence correlates of metastrongyle lungworms in marine mammals
Source: Parasitology. 2023 Oct 20;150(13):1178–91. doi: 10.1017/S0031182023001014 (PMC10801380; doi:10.1017/S0031182023001014)
Supplement: Fischbach and Seguel supplementary material 3 — Fischbach and Seguel supplementary material [file S0031182023001014sup003.docx]

**Supplementary Table 1.** Checklist of all known relationships concerning metastrongyles in marine mammals, complete with information on anatomical ranges documented for each relationship.

| **Host Family** | **Host Species** | **Common Name** | **Parasite Family** | **Parasite species** | **Anatomical Location** | **Reference** |
| --- | --- | --- | --- | --- | --- | --- |
| Balaenopteridae | Megaptera novaeangliae | *Humpback Whale* | Pseudaliidae | *Pharurus alatus* | N/A | (Raga and Balbuena, 1987) |
| Delphinidae | Cephalorhynchus commersonii | *Commerson's Dolphin* | Pseudaliidae | *Halocercus brasiliensis* | Lower respiratory | (Greenwood and Taylor, 1979) |
|  |  |  |  | *Halocercus sp.* | Lower respiratory | (Goodall *et al.*, 1988; Dougnac and Fredes, 2012) |
|  |  |  |  | *Stenurus sp.* | Lower respiratory | (Duignan, 2000) |
|  | Cephalorhynchus hectori | *Hector's dolphin* | Pseudaliidae | *Halocercus sp.* | Lower respiratory | (McKenzie and Blair, 1983; Duignan, 2000) |
|  | Delphinus delphis | *Short-beaked common dolphin* | Pseudaliidae | *Halocercus cryptocephalus* | N/A | (Delyamure, 1968) |
|  |  |  |  | *Halocercus delphini* | Lower respiratory | (Abollo *et al.*, 1998; Gibson *et al.*, 1998; Davison *et al.*, 2013; Pool *et al.*, 2020b, 2021) |
|  |  |  |  | *Halocercus invaginatus* | N/A | (Raga and Balbuena, 1987) |
|  |  |  |  | *Halocercus kleinenbergi* | N/A | (Harris, 1982) |
|  |  |  |  | *Halocercus lagenorhynchi* | Lower respiratory | (Tomo *et al.*, 2010) |
|  |  |  |  | *Halocercus sp.* | Lower respiratory | (Zafra *et al.*, 2015b; Lehnert *et al.*, 2017) |
|  |  |  |  | *Stenurus minor* | N/A | (Raga, 1994) |
|  |  |  |  | *Stenurus ovatus* | Lower respiratory | (Pool *et al.*, 2021) |
|  |  |  |  | *Stenurus sp.* | Lower respiratory | (DÍaz-Delgado *et al.*, 2012) |
|  | Delphinus sp. | *Common dolphin* | Filaroididae | *Parafilaroides sp.* | Lower respiratory | (Stockin *et al.*, 2009) |
|  | Feresa attenuata | *Pygmy killer whale* | Pseudaliidae | *Halocercus brasiliensis* | Lower respiratory | (Guimarães *et al.*, 2015) |
|  |  |  |  | *Halocercus sp.* | Lower respiratory | (Mignucci-Giannoni *et al.*, 1998b) |
|  |  |  |  | *Stenurus globicephalae* | Gastrointestinal, Lower respiratory, Otic apparatus | (Forrester *et al.*, 1980; Mignucci-Giannoni *et al.*, 1998b) |
|  | Globicephala macrorhynchus | *Short-finned pilot whale* | Pseudaliidae | *Halocercus kleinenbergi* | Lower respiratory | (Carvalho *et al.*, 2010) |
|  |  |  |  | *Stenurus globicephalae* | Gastrointestinal, Otic apparatus | (Arnold and Gaskin, 1975; Mignucci-Giannoni *et al.*, 1998b; Carvalho *et al.*, 2010) |
|  |  |  |  | *Stenurus minor* | N/A | (Morales Vela and Olvera Gómez, 1993) |
|  |  |  |  | *Stenurus sp.* | Otic apparatus | (Huertas and Lagueux, 2016) |
|  |  |  |  | *Torynurus convolutus* | N/A | (Dougherty, 1943) |
|  | Globicephala melas | *Long-finned pilot whale* | Pseudaliidae | *Stenurus globicephalae* | Lower respiratory, Otic apparatus | (Cowan, 1967; Arnold and Gaskin, 1975; McManus *et al.*, 1984; Abollo *et al.*, 1998; Gibson *et al.*, 1998; Lehnert *et al.*, 2017; Pool *et al.*, 2021) |
|  |  |  |  | *Torynurus convolutus* | Lower respiratory | (Baylis and Daubney, 1925; Baylis, 1932) |
|  | Grampus griseus | *Risso's dolphin* | Pseudaliidae | *Halocercus delphini* | Lower respiratory | (Pool *et al.*, 2021) |
|  |  |  |  | *Stenurus globicephalae* | Gastrointestinal, Lower respiratory, Otic apparatus | (Arnold and Gaskin, 1975; Fernández *et al.*, 2003; Lehnert *et al.*, 2017; Pool *et al.*, 2021) |
|  |  |  |  | *Stenurus minor* | N/A | (Tomilin, 1967) |
|  | Lagenodelphis hosei | *Fraser's dolphin* | Pseudaliidae | *Stenurus ovatus* | Lower respiratory | (McColl and Obendorf, 1982) |
|  | Lagenorhynchus acutus | *White sided dolphin* | Pseudaliidae | *Halocercus lagenorhynchi* | N/A | (Raga and Balbuena, 1987) |
|  |  |  |  | *Pseudalius inflexus* | N/A | (Evans, 1991) |
|  |  |  |  | *Stenurus globicephalae* | Gastrointestinal, Otic apparatus | (Arnold and Gaskin, 1975; Beverley-Burton, 1978; Rogan *et al.*, 1997; Schick *et al.*, 2020) |
|  |  |  |  | *Torynurus convolutus* | N/A | (Gibson and Harris, 1979) |
|  | Lagenorhynchus albirostris | *White-beaked dolphin* | Pseudaliidae | *Halocercus lagenorhynchi* | Lower respiratory | (Baylis and Daubney, 1925; Gibson and Harris, 1979) |
|  | Lagenorhynchus obscurus | *Dusky dolphin* | Pseudaliidae | *Halocercus sp.* | Gastrointestinal | (von Waerebeek *et al.*, 1993) |
|  |  |  |  | *Stenurus sp.* | Gastrointestinal | (de Castro *et al.*, 2014) |
|  | Lissodelphis borealis | *Northern right whale dolphin* | Pseudaliidae | *Halocercus sp.* | N/A | (Moser and Rhinehart, 1993) |
|  | Orcinus orca | *Killer whale (Orca)* | Pseudaliidae | *Halocercus sp.* | Lower respiratory | (Reckendorf *et al.*, 2018) |
|  | Peponocephala electra | *Melon-headed whale* | Pseudaliidae | *Halocercus sp.* | N/A | (Dailey and Brownell, 1972) |
|  |  |  |  | *Stenurus globicephalae* | Otic apparatus | (Cannon, 1977; Mignucci-Giannoni *et al.*, 1998a; b; Carvalho *et al.*, 2010) |
|  | Pseudorca crassidens | *False killer whale* | Pseudaliidae | *Stenurus auditivus* | N/A | (Zam *et al.*, 1971) |
|  |  |  |  | *Stenurus globicephalae* | Otic apparatus | (Zylber *et al.*, 2002) |
|  | Sotalia fluviatilis | *Tucuxi* | Pseudaliidae | *Halocercus brasiliensis* | Lower respiratory | (Santos *et al.*, 1996; Di Beneditto and Ramos, 2004; Melo *et al.*, 2006; Rodrigues *et al.*, 2018) |
|  | Sotalia guianensis | *Guiana dolphin* | Pseudaliidae | *Halocercus brasiliensis* | Gastrointestinal, Lower respiratory | (Marigo *et al.*, 2010; Carvalho *et al.*, 2010; Guimarães *et al.*, 2013, 2015; Domiciano *et al.*, 2016; Groch *et al.*, 2018, 2020b; Carvalho Demarque *et al.*, 2020) |
|  |  |  |  | *Halocercus sp.* | Lower respiratory | (Carvalho *et al.*, 2010) |
|  | Sousa chinensis | *Indo-Pacific humpback dolphin* | Pseudaliidae | *Halocercus pingi* | Lower respiratory | (Parsons and Jefferson, 2000; Parsons *et al.*, 2001) |
|  | Sousa plumbea | *Indian Ocean humpback dolphin* | Pseudaliidae | *Halocercus sp.* | Lower respiratory | (Lane *et al.*, 2014) |
|  | Stenella attenuata | *Pantropical spotted dolphin* | Pseudaliidae | *Halocercus delphini* | Lower respiratory | (Dailey and Perrin, 1973) |
|  |  |  |  | *Halocercus sp.* | Lower respiratory | (Oliveira *et al.*, 2011) |
|  | Stenella clymene | *Clymene dolphin* | Pseudaliidae | *Halocercus brasiliensis* | Lower respiratory | (Carvalho *et al.*, 2010; Guimarães *et al.*, 2015) |
|  |  |  |  | *Halocercus delphini* | Lower respiratory, Upper respiratory | (Aguilar-Aguilar *et al.*, 2010; Hernández-Orts *et al.*, 2021) |
|  |  |  |  | *Halocercus sp.* | Lower respiratory | (Carvalho *et al.*, 2010) |
|  | Stenella coeruleoalba | *Striped dolphin* | Pseudaliidae | *Halocercus brasiliensis* | Lower respiratory | (Rosas *et al.*, 2002) |
|  |  |  |  | *Halocercus delphini* | Lower respiratory | (Lehnert *et al.*, 2017; Pool *et al.*, 2020a; b, 2021; Terracciano *et al.*, 2020) |
|  |  |  |  | *Halocercus lagenorhynchi* | Lower respiratory | (Ross and Bass, 1971; Gibson *et al.*, 1998; Oliveira *et al.*, 2011; Terracciano *et al.*, 2020) |
|  |  |  |  | *Halocercus sp.* | Lower respiratory | (Abollo *et al.*, 1998; Carvalho *et al.*, 2010; Zafra *et al.*, 2015b; Lehnert *et al.*, 2017; Vargas-Castro *et al.*, 2020; García de los Ríos y Loshuertos *et al.*, 2021) |
|  |  |  |  | *Pharurus sp.* | N/A | (García de los Ríos y Loshuertos *et al.*, 2021) |
|  |  |  |  | *Pseudalius sp.* | N/A | (García de los Ríos y Loshuertos *et al.*, 2021) |
|  |  |  |  | *Stenurus minor* | Gastrointestinal | (Aytemiz *et al.*, 2012) |
|  |  |  |  | *Stenurus ovatus* | Lower respiratory | (Pool *et al.*, 2020a, 2021) |
|  |  |  |  | *Stenurus sp.* | N/A | (García de los Ríos y Loshuertos *et al.*, 2021) |
|  | Stenella frontalis | *Spotted dolphin* | Pseudaliidae | *Halocercus delphini* | N/A | (Perrin *et al.*, 1994) |
|  |  |  |  | *Halocercus lagenorhynchi* | N/A | (Perrin *et al.*, 1987) |
|  |  |  |  | *Halocercus sp.* | Lower respiratory | (Zafra *et al.*, 2015b) |
|  |  |  |  | *Pseudalius sp.* | Lower respiratory | (Zafra *et al.*, 2015b) |
|  | Stenella longirostris | *Spinner dolphin* | Pseudaliidae | *Halocercus brasiliensis* | Lower respiratory | (Carvalho *et al.*, 2010; Domiciano *et al.*, 2016) |
|  |  |  |  | *Halocercus delphini* | Lower respiratory | (Dailey and Perrin, 1973) |
|  |  |  |  | *Halocercus sp.* | Lower respiratory | (Carvalho *et al.*, 2010; Oliveira *et al.*, 2011) |
|  | Steno bredanensis | *Rough-toothed dolphin* | Pseudaliidae | *Stenurus sp.* | Otic apparatus | (Ewing *et al.*, 2020) |
|  | Tursiops aduncus | *Indo-Pacific bottlenose dolphin* | Pseudaliidae | *Halocercus lagenorhynchi* | Lower respiratory | (Tomo *et al.*, 2010) |
|  |  |  |  | *Halocercus sp.* | Lower respiratory | (Stephens *et al.*, 2014; Lane *et al.*, 2014) |
|  |  |  |  | *Stenurus ovatus* | Lower respiratory | (Tomo *et al.*, 2010) |
|  | Tursiops truncatus | *Common bottlenose dolphin* | Pseudaliidae | *Halocercus cryptocephalus* | Lower respiratory | (Bowie, 1984; Fauquier *et al.*, 2009) |
|  |  |  |  | *Halocercus delphini* | Lower respiratory | (Abollo *et al.*, 1998; Terracciano *et al.*, 2020; Pool *et al.*, 2021) |
|  |  |  |  | *Halocercus invaginatus* | N/A | (Birkun and Krivokhizhin, 1996) |
|  |  |  |  | *Halocercus lagenorhynchi* | Lower respiratory | (Woodard *et al.*, 1969; Dailey *et al.*, 1991b; Lipscomb *et al.*, 1996; Mazzariol *et al.*, 2007; McFee and Lipscomb, 2009; Fauquier *et al.*, 2009) |
|  |  |  |  | *Halocercus sp.* | Lower respiratory | (Sierra *et al.*, 2014; Seguel *et al.*, 2020b) |
|  |  |  |  | *Pharurus alatus* | Lower respiratory | (Tomo *et al.*, 2010) |
|  |  |  |  | *Stenurus minor* | Lower respiratory | (Birincioğlu *et al.*, 2017) |
|  |  |  |  | *Stenurus ovatus* | Lower respiratory | (Bowie, 1984; Kumazawa *et al.*, 2006; Kuwamura *et al.*, 2007; Tomo *et al.*, 2010; Terracciano *et al.*, 2020; Pool *et al.*, 2021) |
|  |  |  |  | *Stenurus sp.* | Lower respiratory | (Sierra *et al.*, 2014) |
| Iniidae | Inia geoffrensis | *Amazon river dolphin* | Pseudaliidae | *Halocercus brasiliensis* | Lower respiratory | (Rodrigues *et al.*, 2018) |
|  |  |  |  | *Halocercus sp.* | N/A | (Dailey and Brownell, 1972) |
| Kogiidae | Kogia breviceps | *Pygmy sperm whale* | Pseudaliidae | *Halocercus sp.* | Lower respiratory | (Carvalho *et al.*, 2010) |
| Monodontidae | Delphinapterus leucas | *Beluga whale* | Pseudaliidae | *Halocercus monoceris* | Cardiovascular, Lower respiratory, Upper respiratory | (Measures *et al.*, 1995) |
|  |  |  |  | *Halocercus taurica* | Cardiovascular, Lower respiratory, Upper respiratory | (Measures *et al.*, 1995) |
|  |  |  |  | *Pharurus pallasii* | Central Nervous System, Lower respiratory, Otic apparatus | (Babero and Thomas, 1960; Arnold and Gaskin, 1975; Kenyon and Kenyon, 1977; Wazura *et al.*, 1986; Measures *et al.*, 1995; Houde *et al.*, 2003) |
|  |  |  |  | *Stenurus arctomarinus* | Cardiovascular, Lower respiratory, Upper respiratory | (Arnold and Gaskin, 1975; Measures *et al.*, 1995; Burek-Huntington *et al.*, 2015) |
|  |  |  |  | *Stenurus minor* | N/A | (Kühn, 1829) |
|  | Monodon monoceros | *Narwhal* | Pseudaliidae | *Halocercus monoceris* | Lower respiratory | (Webster *et al.*, 1973; MacNeill *et al.*, 1975) |
|  |  |  |  | *Pharurus alatus* | N/A | (Arnold and Gaskin, 1975) |
| Otariidae | Arctocephalus australis | *South american fur seal* | Filaroididae | *Parafilaroides sp.* | Lower respiratory | (Jacobus *et al.*, 2016) |
|  |  |  |  | *Parafilaroides normani* | Lower respiratory | (Echenique *et al.*, 2020) |
|  | Arctocephalus forsteri | *New zealand fur seal* | Filaroididae | *Parafilaroides normani* | Lower respiratory | (Dailey, 2009) |
|  | Arctocephalus gazella | *Antarctic fur seal* | Filaroididae | *Parafilaroides normani* | Lower respiratory | (Jacobus *et al.*, 2016) |
|  | Arctocephalus philippii | *Guadalupe Fur seal* | Filaroididae | *Parafilaroides decorus* | Cardiovascular, Lower respiratory | (Seguel *et al.*, 2018a; Williams *et al.*, 2020) |
|  | Arctocephalus pusillus | *Australian fur seal* | Filaroididae | *Parafilaroides normani* | Lower respiratory | (Dailey, 2009; Jabbar *et al.*, 2014) |
|  | Arctocephalus tropicalis | *Sub-antarctic fur seal* | Filaroididae | *Parafilaroides sp.* | Lower respiratory | (Reisfeld *et al.*, 2019) |
|  |  |  |  | *Parafilaroides normani* | Lower respiratory | (Dailey, 2009) |
|  | Callorhinus ursinus | *Northern fur seal* | Filaroididae | *Parafilaroides decorus* | N/A | (Gerber *et al.*, 1993) |
|  | Eumetopias jubatus | *Stellar sea lion* | Filaroididae | *Parafilaroides decorus* | Lower respiratory | (Stroud, 1978; Stroud and Roffe, 1979) |
|  |  |  |  | *Parafilaroides nanus* | N/A | (Dougherty and Herman, 1947) |
|  |  |  |  | *Parafilaroides prolificus* | N/A | (Dougherty and Herman, 1947) |
|  | Neophoca cinerea | *Australian sea lion* | Filaroididae | *Parafilaroides sp.* | Lower respiratory | (Nicholson and Fanning, 1981) |
|  | Otaria flavescens | *South American sea lion* | Crenosomatidae | *Otostrongylus circumlitus* | N/A | (Ebmer *et al.*, 2020) |
|  |  |  |  | *Otostrongylus sp.* | N/A | (Hermosilla *et al.*, 2016) |
|  |  |  | Filaroididae | *Parafilaroides sp.* | N/A | (Hermosilla *et al.*, 2016) |
|  | Phocarctos hookeri | *New zealand sea lion* | Filaroididae | *Parafilaroides decorus* | N/A | (Duignan, 2000) |
|  | Zalophus californianus | *Californian sea lion* | Crenosomatidae | *Otostrongylus circumlitus* | Cardiovascular, Lower respiratory | (Kelly *et al.*, 2005; Goldstein *et al.*, 2011) |
|  |  |  | Filaroididae | *Parafilaroides sp.* | Lower respiratory | (Gulland *et al.*, 2012) |
|  |  |  |  | *Parafilaroides decorus* | Lower respiratory | (Johnston and Ridgway, 1969; Dailey, 1970b; Fleischman and Squire, 1970; Migaki *et al.*, 1971; Sweeney and Gilmartin, 1974; Stroud, 1978; Gerber *et al.*, 1993; Haulena and Gulland, 2001; Fauquier *et al.*, 2004; Greig *et al.*, 2005; Dennison *et al.*, 2007; Evans, 2011; Kuzmina *et al.*, 2018; Williams *et al.*, 2020; Liu *et al.*, 2021) |
|  | Zalophus wollebaeki | *Galapagos sea lion* | Crenosomatidae | *Otostrongylus sp.* | N/A | (Walden *et al.*, 2018) |
|  |  |  | Filaroididae | *Parafilaroides sp.* | N/A | (Walden *et al.*, 2018) |
| Phocidae | Cystophora cristata | *Hooded seal* | Crenosomatidae | *Otostrongylus circumlitus* | Lower respiratory | (Lucas *et al.*, 2003) |
|  |  |  | Filaroididae | *Parafilaroides gymnurus* | Lower respiratory | (Lucas *et al.*, 2003) |
|  | Erignathus barbatus | *Bearded seal* | Crenosomatidae | *Otostrongylus circumlitus* | N/A | (Antonelis *et al.*, 1994) |
|  |  |  | Filaroididae | *Parafilaroides sp.* | Cardiovascular, Lower respiratory | (Walden *et al.*, 2020) |
|  |  |  |  | *Parafilaroides gymnurus* | Lower respiratory | (Gosselin *et al.*, 1998; Walden *et al.*, 2020) |
|  | Halichoerus grypus | *Grey seal* | Crenosomatidae | *Otostrongylus circumlitus* | Cardiovascular, Lower respiratory | (Gosselin *et al.*, 1998; Ulrich *et al.*, 2015; Barnett *et al.*, 2019a, 2021) |
|  |  |  |  | *Otostrongylus sp.* | Lower respiratory | (Baker, 1987) |
|  |  |  | Filaroididae | *Parafilaroides sp.* | Lower respiratory | (Baker, 1987; Barnett *et al.*, 2019a, 2021) |
|  |  |  |  | *Parafilaroides gymnurus* | Lower respiratory | (Gosselin and Measures, 1997; Gosselin *et al.*, 1998; Ulrich *et al.*, 2015) |
|  |  |  |  | *Parafilaroides hispidus* | Lower respiratory | (Gosselin and Measures, 1997; Gosselin *et al.*, 1998) |
|  | Histriophoca fasciata | *Ribbon seal* | Crenosomatidae | *Otostrongylus circumlitus* | N/A | (Walden *et al.*, 2020) |
|  |  |  |  | *Otostrongylus sp.* | N/A | (Walden *et al.*, 2020) |
|  |  |  | Filaroididae | *Parafilaroides gymnurus* | Lower respiratory | (Walden *et al.*, 2020) |
|  | Hydrurga leptonyx | *Leopard seal* | Filaroididae | *Parafilaroides hydrurgae* | Lower respiratory | (Mawson, 1953) |
|  | Mirounga angustirostris | *Northern elephant seal* | Crenosomatidae | *Otostrongylus circumlitus* | Cardiovascular,  Gastrointestinal,  Lower respiratory | (Stroud, 1978; Stroud and Roffe, 1979; Gulland *et al.*, 1997a; Elson-Riggins *et al.*, 2001; Fauquier *et al.*, 2003; Elson-Riggins *et al.*, 2004; Colegrove *et al.*, 2005; Kaye *et al.*, 2016; Sheldon *et al.*, 2017; Kaye *et al.*, 2017; Sheldon *et al.*, 2019) |
|  |  |  |  | *Otostrongylus sp.* | N/A | (Gerber *et al.*, 1993) |
|  |  |  | Filaroididae | *Parafilaroides sp.* | Lower respiratory | (Stroud, 1978; Stroud and Roffe, 1979) |
|  |  |  |  | *Parafilaroides decorus* | N/A | (Gerber *et al.*, 1993) |
|  |  |  |  | *Parafilaroides measuresae* | Lower respiratory | (Dailey, 2006b) |
|  | Pagophilus groenlandicus | *Harp seal* | Crenosomatidae | *Otostrongylus circumlitus* | Lower respiratory, Upper respiratory | (Lucas *et al.*, 2003; Piché *et al.*, 2010) |
|  |  |  | Filaroididae | *Parafilaroides gymnurus* | Lower respiratory | (Gosselin and Measures, 1997; Gosselin *et al.*, 1998; Lucas *et al.*, 2003) |
|  | Phoca largha | *Spotted seal* | Crenosomatidae | *Otostrongylus circumlitus* | N/A | (Delyamure *et al.*, 1984; Walden *et al.*, 2020) |
|  |  |  | Filaroididae | *Parafilaroides gymnurus* | Lower respiratory | (Delyamure *et al.*, 1984; Walden *et al.*, 2020) |
|  | Phoca vitulina | *Harbour seal* | Crenosomatidae | *Otostrongylus circumlitus* | Cardiovascular, Lower respiratory | (Dunn and Wolke, 1976; Kennedy *et al.*, 1989; Claussen *et al.*, 1991; Borgsteede *et al.*, 1991; Gerber *et al.*, 1993; Gosselin *et al.*, 1998; Elson-Riggins *et al.*, 2001, 2004; Fouchier *et al.*, 2001; Vercruysse *et al.*, 2003; Colegrove *et al.*, 2005; McKnight *et al.*, 2005; Lehnert *et al.*, 2007, 2010; Leidenberger and Boström, 2009; Lambourn *et al.*, 2013; Osinga *et al.*, 2015; Ulrich *et al.*, 2015, 2016; Kroese *et al.*, 2018) |
|  |  |  |  | *Otostrongylus sp.* | N/A | (Herreman *et al.*, 2011) |
|  |  |  | Filaroididae | *Parafilaroides sp.* | Lower respiratory | (Garner *et al.*, 1997; Prenger-Berninghoff *et al.*, 2008; Herreman *et al.*, 2011; Lambourn *et al.*, 2013; Rhyan *et al.*, 2018b) |
|  |  |  |  | *Parafilaroides decorus* | Lower respiratory | (Greig *et al.*, 2014) |
|  |  |  |  | *Parafilaroides gullandae* | Lower respiratory | (Dailey, 2006b) |
|  |  |  |  | *Parafilaroides gymnurus* | Lower respiratory, Reproductive | (Schumacher *et al.*, 1990; Claussen *et al.*, 1991; Borgsteede *et al.*, 1991; Gosselin and Measures, 1997; Gosselin *et al.*, 1998; Fouchier *et al.*, 2001; Vercruysse *et al.*, 2003; Lehnert *et al.*, 2007, 2010; Osinga *et al.*, 2015; Ulrich *et al.*, 2015; Elson-Riggins *et al.*, 2020) |
|  | Pusa caspica | *Caspian seal* | Filaroididae | *Parafilaroides caspicus* | N/A | (Kurochkin and Zablotsky, 1958) |
|  | Pusa hispida | *Ringed seal* | Crenosomatidae | *Otostrongylus circumlitus* | Lower respiratory | (Onderka, 1989; Measures and Gosselin, 1994; Bergeron *et al.*, 1997a; b; Walden *et al.*, 2020) |
|  |  |  | Filaroididae | *Parafilaroides sp.* | Lower respiratory | (Walden *et al.*, 2020) |
|  |  |  |  | *Parafilaroides gymnurus* | Gastrointestinal, Lower respiratory | (Measures and Gosselin, 1994; Gosselin and Measures, 1997; Gosselin *et al.*, 1998; Walden *et al.*, 2020) |
|  |  |  |  | *Parafilaroides hispidus* | Lower respiratory | (Kennedy, 1986; Onderka, 1989; Gosselin and Measures, 1997; Gosselin *et al.*, 1998) |
|  | Pusa sibirica | *Baikal seal* | Crenosomatidae | *Otostrongylus circumlitus* | Lower respiratory | (Suvorova and Prokushina, 2021) |
|  |  |  | Filaroididae | *Parafilaroides gymnurus* | Lower respiratory | (Kontrimavichus and Delyamure, 1979; Suvorova and Prokushina, 2021) |
| Phocoenidae | Neophocaena asiaeorientalis | *Narrow-ridged finless porpoise* | Pseudaliidae | *Halocercus pingi* | Lower respiratory, Reproductive | (Shiozaki and Amano, 2017) |
|  |  |  |  | *Halocercus sp.* | Lower respiratory | (Wan *et al.*, 2017; Gui *et al.*, 2018) |
|  |  |  |  | *Halocercus sunameri* | Lower respiratory | (Shiozaki and Amano, 2017) |
|  |  |  |  | *Halocercus taurica* | Lower respiratory | (Shiozaki and Amano, 2017) |
|  |  |  |  | *Pharurus asiaeorientalis* | Lower respiratory, Otic apparatus | (Shiozaki and Amano, 2017) |
|  |  |  |  | *Pharurus sunameri* | Lower respiratory, Otic apparatus | (Shiozaki and Amano, 2017) |
|  |  |  |  | *Stenurus nanjingensis* | Otic apparatus | (Shiozaki and Amano, 2017) |
|  | Neophocaena phocaenoides | *Indo-Pacific finless porpoise* | Crenosomatidae | *Otostrongylus circumlitus* | N/A | (Yu *et al.*, 2009) |
|  |  |  | Pseudaliidae | *Halocercus pingi* | Lower respiratory | (Wu, 1929; Parsons and Jefferson, 2000; Parsons *et al.*, 2001) |
|  |  |  |  | *Halocercus sp.* | Lower respiratory | (Gui *et al.*, 2018) |
|  |  |  |  | *Halocercus sunameri* | N/A | (Yamaguti, 1951) |
|  |  |  |  | *Halocercus taurica* | N/A | (Kuramochi *et al.*, 2000) |
|  |  |  |  | *Pharurus asiaeorientalis* | N/A | (Petter and Pilleri, 1982) |
|  |  |  |  | *Pharurus sunameri* | N/A | (Yamaguti, 1951) |
|  |  |  |  | *Stenurus auditivus* | N/A | (Hsü and Hoeppli, 1933) |
|  |  |  |  | *Stenurus nanjingensis* | N/A | (Tao, 1983) |
|  | Phocoena dioptrica | *Spectacled porpoise* | Pseudaliidae | *Stenurus minor* | Otic apparatus | (Lehnert *et al.*, 2017) |
|  | Phocoena phocoena | *Harbour Porpoise* | Pseudaliidae | *Halocercus invaginatus* | Cardiovascular, Lower respiratory, Upper respiratory | (Smith and Threlfall, 1973; Andersen, 1974; Arnold and Gaskin, 1975; Dailey and Stroud, 1978; Stroud and Roffe, 1979; Kastelein and Lavaleije, 1992; Moser and Rhinehart, 1993; Balbuena *et al.*, 1994; Abollo *et al.*, 1998; Gibson *et al.*, 1998; Jepson *et al.*, 2000; Rogan *et al.*, 2001; Lehnert *et al.*, 2005, 2010, 2014; Siebert *et al.*, 2006, 2020; Veryeri, 2012; Pekmezci̇ *et al.*, 2013) |
|  |  |  |  | *Halocercus kirbyi* | N/A | (Dougherty, 1944) |
|  |  |  |  | *Halocercus sp.* | Lower respiratory | (Smith and Threlfall, 1973; Wunschimann *et al.*, 2001; Jauniaux *et al.*, 2002; Dagleish *et al.*, 2008; Valderrama Vasquez *et al.*, 2008; Fenton *et al.*, 2017; van Elk *et al.*, 2019) |
|  |  |  |  | *Halocercus taurica* | Lower respiratory | (Arnold and Gaskin, 1975; Gibson *et al.*, 1998; Jepson *et al.*, 2000; Rogan *et al.*, 2001; Pekmezci̇ *et al.*, 2013) |
|  |  |  |  | *Pharurus dalli* | Lower respiratory, Otic apparatus | (Smith and Threlfall, 1973; Colón-Llavina *et al.*, 2019) |
|  |  |  |  | *Pharurus sp.* | N/A | (Smith and Threlfall, 1973) |
|  |  |  |  | *Pseudalius inflexus* | Cardiovascular,  Gastrointestinal, Lower respiratory, Otic apparatus | (Andersen, 1974; Arnold and Gaskin, 1975; Kastelein and Lavaleije, 1992; Balbuena *et al.*, 1994; Brosens *et al.*, 1996; Szefer *et al.*, 1998; Gibson *et al.*, 1998; Wünschmann *et al.*, 1999; Jepson *et al.*, 2000; Siebert *et al.*, 2001, 2006, 2013, 2020; Wunschimann *et al.*, 2001; Rogan *et al.*, 2001; Jauniaux *et al.*, 2002; Lehnert *et al.*, 2005, 2010; Dawson *et al.*, 2008; Dagleish *et al.*, 2008; Valderrama Vasquez *et al.*, 2008; Davison *et al.*, 2010a; b; Seibel *et al.*, 2010; Fenton *et al.*, 2017; van Elk *et al.*, 2019; Gabel *et al.*, 2021) |
|  |  |  |  | *Stenurus minor* | Gastrointestinal, Lower respiratory, Otic apparatus, Upper Respiratory | (Andersen, 1974; Arnold and Gaskin, 1975; Dailey and Stroud, 1978; Stroud and Roffe, 1979; Faulkner, 1995; Brosens *et al.*, 1996; Faulkner *et al.*, 1998; Gibson *et al.*, 1998; Wünschmann *et al.*, 1999; Jepson *et al.*, 2000; Siebert *et al.*, 2001, 2006, 2013, 2020; Wunschimann *et al.*, 2001; Rogan *et al.*, 2001; Jauniaux *et al.*, 2002; Kijewska *et al.*, 2003; Lehnert *et al.*, 2005, 2010, 2014; Prahl *et al.*, 2008; Valderrama Vasquez *et al.*, 2008; Seibel *et al.*, 2010; Pekmezci̇ *et al.*, 2013; Fenton *et al.*, 2017; Morell *et al.*, 2017; Wohlsein *et al.*, 2019b; van Elk *et al.*, 2019; Colón-Llavina *et al.*, 2019; Gabel *et al.*, 2021) |
|  |  |  |  | *Stenurus sp.* | Lower respiratory | (Fenton *et al.*, 2017) |
|  |  |  |  | *Torynurus convolutus* | Cardiovascular,  Gastrointestinal, Lower respiratory,  Otic apparatus, Upper respiratory | (Arnold and Gaskin, 1975; Dailey and Stroud, 1978; Kastelein and Lavaleije, 1992; Balbuena *et al.*, 1994; Brosens *et al.*, 1996; Faulkner *et al.*, 1998; Gibson *et al.*, 1998; Wünschmann *et al.*, 1999; Jepson *et al.*, 2000; Siebert *et al.*, 2001, 2006, 2013, 2020; Wunschimann *et al.*, 2001; Rogan *et al.*, 2001; Jauniaux *et al.*, 2002; Lehnert *et al.*, 2005, 2010; Valderrama Vasquez *et al.*, 2008; Davison *et al.*, 2010a; Seibel *et al.*, 2010; Fenton *et al.*, 2017; van Elk *et al.*, 2019) |
|  | Phocoena spinipinnis | *Burmeister's porpoise* | Pseudaliidae | *Halocercus sp.* | Lower respiratory | (Reyes and van Waerebeek, 1995) |
|  |  |  |  | *Pseudalius inflexus* | Lower respiratory | (Corcuera *et al.*, 1995; Reyes and van Waerebeek, 1995; Alvarado-Rybak *et al.*, 2020b) |
|  |  |  |  | *Stenurus australis* | Lower respiratory, Otic apparatus | (Torres *et al.*, 1994; Alvarado-Rybak *et al.*, 2020b) |
|  |  |  |  | *Stenurus minor* | Otic apparatus | (Corcuera *et al.*, 1995) |
|  |  |  |  | *Stenurus sp.* | Otic apparatus | (Reyes and van Waerebeek, 1995) |
|  | Phocoenoides dalli | *Dall’s porpoise* | Pseudaliidae | *Halocercus dalli* | Lower respiratory | (Conlogue *et al.*, 1985) |
|  |  |  |  | *Halocercus kirbyi* | N/A | (Dougherty, 1944) |
|  |  |  |  | *Halocercus sp.* | Lower respiratory | (Migaki *et al.*, 1971) |
|  |  |  |  | *Pharurus dalli* | Otic apparatus | (Arnold and Gaskin, 1975; Dailey and Walker, 1978; Kuramochi *et al.*, 1990) |
|  |  |  |  | *Pharurus sp.* | N/A | (Walker, 1975) |
|  |  |  |  | *Stenurus minor* | Lower respiratory, Otic apparatus | (Johnston and Ridgway, 1969; Migaki *et al.*, 1971; Dailey and Walker, 1978) |
|  |  |  |  | *Stenurus truei* | Otic apparatus | (Kuramochi *et al.*, 1990) |
|  |  |  |  | *Stenurus yamagutii* | Otic apparatus | (Kuramochi *et al.*, 1990) |
| Physteridae | Physeter macrocephalus | *Sperm whale* | Pseudaliidae | *Stenurus ovatus* | N/A | (Raga and Balbuena, 1987) |
| Ziphiidae | Berardius bairdii | *Baird's beaked whale* | Pseudaliidae | *Halocercus hyperoodoni* | N/A | (Delyamure, 1968) |
|  | Mesoplodon sp. | *Unidentified whale* | Pseudaliidae | *Halocercus sp.* | Lower respiratory | (Moser and Rhinehart, 1993) |
|  | Mesoplodon stejnegeri | *Stejneger's beaked whale* | Pseudaliidae | *Halocercus sp.* | Lower respiratory | (Savage *et al.*, 2021) |

**References**

**Abollo, E., López, A., Gestal, C., Benavente, P. and Pascual, S.** (1998). Macroparasites in cetaceans stranded on the northwestern Spanish Atlantic coast. *Diseases of Aquatic Organisms* **32**, 227–231. doi: 10.3354/dao032227.

**Aguilar-Aguilar, R., Delgado-Estrella, A. and Moreno-Navarrete, R.** (2010). New host report for nematodes in a stranded short-snouted spinner dolphin Stenella clymene (Cetacea: Delphinidae) from the Mexican Caribbean coast. *Helminthologia* **47**, 136–138. doi: 10.2478/s11687-010-0020-0.

**Alvarado-Rybak, M., Toro, F., Abarca, P., Paredes, E., Español-Jiménez, S. and Seguel, M.** (2020). Pathological Findings in Cetaceans Sporadically Stranded Along the Chilean Coast. *Frontiers in Marine Science* **7**, 684. doi: 10.3389/fmars.2020.00684.

**Andersen, S. H.** (1974). A Typical Case History of the Net-Caught Harbour Porpose. *Aquatic Mammals* **2**, 1–6.

**Antonelis, G. A., Melin, S. R. and Bukhtiyarov, Y. A.** (1994). Early Spring Feeding Habits of Bearded Seals (Erignathus Barbatus) in the Central Bering Sea, 1981. *ARCTIC* **47**, 74–79. doi: 10.14430/arctic1274.

**Arnold, P. W. and Gaskin, D. E.** (1975). Lungworms (Metastrongyloidea: Pseudaliidae) of harbor porpoise Phocoena phocoena (L. 1758). *Canadian Journal of Zoology* **53**, 713–735. doi: 10.1139/z75-087.

**Aytemiz, I., Dede, A., Danyer, E. and Tonay, A. M.** (2012). Morphological identification of parasites found in the stomach contents of bycaught striped dolphins (Stenella coeruleoalba) from Turkish Eastern Mediterranean Sea coast. *Journal of the Black Sea / Mediterranean Environment* **18**, 238–245.

**Babero, B. B. and Thomas, L. J.** (1960). A Record of Pharurus oserkaiae (Skrjabin, 1942) in an Alaskan Whale. *The Journal of Parasitology* **46**, 726. doi: 10.2307/3275519.

**Baker, J. R.** (1987). Causes of mortality and morbidity in wild juvenile and adult grey seals (Halichoerus grypus). *British Veterinary Journal* **143**, 203–220. doi: 10.1016/0007-1935(87)90083-2.

**Balbuena, J. A., Aspholm, P. E., Andersen, K. I. and Bjørge, A.** (1994). Lung-worms (Nematoda: Pseudaliidae) of harbour porpoises ( *Phocoena phocoena* ) in Norwegian waters: patterns of colonization. *Parasitology* **108**, 343–349. doi: 10.1017/S0031182000076186.

**Barnett, J. E. F., Bexton, S., Fraija-Fernández, N., Chooneea, D. and Wessels, M. E.** (2019). Novel Pulmonary Vasculitis with Splendore–Hoeppli Reaction in Grey Seals (Halichoerus grypus) Associated with Otostrongylus circumlitus Infection. *Journal of Comparative Pathology* **173**, 83–91. doi: 10.1016/j.jcpa.2019.10.009.

**Barnett, J., Allen, R., Astley, K., Whitehouse, F. and Wessels, M.** (2021). Pathology of grey seals Halichoerus grypus in southwest England including pups in early rehabilitation. *Diseases of Aquatic Organisms* **145**, 35–50. doi: 10.3354/dao03600.

**Baylis, H. A.** (1932). A list of worms parasitic in Cetacea. *Discovery Reports* **6**, 393–418.

**Baylis, H. A. and Daubney, R.** (1925). A Revision of the Lung-Worms of Cetacea. *Parasitology* **17**, 201–215. doi: 10.1017/S0031182000004595.

**Bergeron, E., Huot, J. and Measures, L. N.** (1997a). Experimental transmission of Otostrongylus circumlitus (Railliet, 1899) (Metastrongyloidea: Crenosomatidae), a lungworm of seals in eastern arctic Canada. *Canadian Journal of Zoology* **75**, 1364–1371. doi: 10.1139/z97-762.

**Bergeron, E., Measures, L. N. and Huot, J.** (1997b). Lungworm (Otostrongylus circumlitus) infections in ringed seals (Phoca hispida) from eastern Arctic Canada. *Canadian Journal of Fisheries and Aquatic Sciences* **54**, 2443–2448. doi: 10.1139/f97-153.

**Beverley-Burton, M.** (1978). Helminths of the Alimentary Tract from a Stranded Herd of the Atlantic White-Sided Dolphin, *Lagenorhynchus acutus*. *Journal of the Fisheries Research Board of Canada* **35**, 1356–1359. doi: 10.1139/f78-211.

**Birincioğlu, S. S., Aypak, S., Avci, H., Birincioğlu, B., İPek, E. and Akkoç, A. N.** (2017). Şişe Burunlu Bir Yunusta (Tursiops truncatus) Patolojik ve Parazitolojik İncelemeler. *Kafkas Universitesi Veteriner Fakultesi Dergisi*. doi: 10.9775/kvfd.2017.18016.

**Birkun, A. A. Jr. and Krivokhizhin, S. V.** (1996). Present state and causes of the Black Sea cetacean populations suppression. Parts I and II. *Vestnik Zoologii* **3**, 36–42.

**Borgsteede, F. H. M., Bus, H. G. J., Verplanke, J. A. W. and van Burg, W. P. J.** (1991). Endoparasitic helminths of the harbour seal, Phoca vitulina, in the Netherlands. *Netherlands Journal of Sea Research* **28**, 247–250. doi: 10.1016/0077-7579(91)90022-S.

**Bowie, J. Y.** (1984). Parasites from an Atlantic bottle-nose dolphin ( *Tursiops truncatus* ), and a revised checklist of parasites of this host. *New Zealand Journal of Zoology* **11**, 395–398. doi: 10.1080/03014223.1984.10428253.

**Brosens, L., Jauniaux, T., Siebert, U., Benke, H. and Coignoul, F.** (1996). Observations on the helminths of harbour porpoises (Phocoena phocoena) and common guillemots (Uria aalge) from the Belgian and German coasts. *Veterinary Record* **139**, 254–257. doi: 10.1136/vr.139.11.254.

**Burek-Huntington, K., Dushane, J., Goertz, C., Measures, L., Romero, C. and Raverty, S.** (2015). Morbidity and mortality in stranded Cook Inlet beluga whales Delphinapterus leucas. *Diseases of Aquatic Organisms* **114**, 45–60. doi: 10.3354/dao02839.

**Cannon, L. R. G.** (1977). Some aspects of the biology of Peponocephala electra (Cetacea: Delphinidae). II. Parasites. *Marine and Freshwater Research* **28**, 717–722.

**Carvalho, V. L., Bevilaqua, C. M. L., Iñiguez, A. M., Mathews-Cascon, H., Ribeiro, F. B., Pessoa, L. M. B., de Meirelles, A. C. O., Borges, J. C. G., Marigo, J., Soares, L. and de Lima Silva, F. J.** (2010). Metazoan parasites of cetaceans off the northeastern coast of Brazil. *Veterinary Parasitology* **173**, 116–122. doi: 10.1016/j.vetpar.2010.06.023.

**Carvalho Demarque, I. de O., Rodrigues de Oliveira, F. C., da Silveira, L. S., Barbosa, L. A. and Ederli, N. B.** (2020). The Lungworm, Halocercus brasiliensis (Nematoda: Pseudaliidae), from Guiana Dolphins Sotalia guianensis from Brazil with Pathological Findings. *Journal of Parasitology* **106**, 254. doi: 10.1645/19-77.

**Claussen, D., Strauss, V., Ising, S., Jäger, M., Schnieder, T. and Stoye, M.** (1991). The Helminth Fauna from the Common Seal (Phoca vitulina vitulina, Linné, 1758) of the Wadden Sea in Lower Saxony*: Part 2: Nematodes. *Journal of Veterinary Medicine, Series B* **38**, 649–656. doi: 10.1111/j.1439-0450.1991.tb00924.x.

**Colegrove, K. M., Greig, D. J. and Gulland, F. M. D.** (2005). Causes of Live Strandings of Northern Elephant Seals (Mirounga angustirostris) and Pacific Harbor Seals (Phoca vitulina) Along the Central California Coast, 1992-2001. *Aquatic Mammals* **31**, 1–10. doi: 10.1578/AM.31.1.2005.1.

**Colón-Llavina, M. M., Mattiucci, S., Nascetti, G., Harvey, J. T., Williams, E. H. and Mignucci-Giannoni, A. A.** (2019). Some Metazoan Parasites from Marine Mammals Stranded in California. *Pacific Science* **73**, 461. doi: 10.2984/73.4.3.

**Conlogue, G. J., Ogden, J. A. and Foreyt, W. J.** (1985). Parasites of the Dall’s porpoise (Phocoenoides dalli True). *Journal of Wildlife Diseases* **21**, 160–166. doi: 10.7589/0090-3558-21.2.160.

**Corcuera, J., Monzon, F., Anguilar, A., Borrell, A. and Raga, J. A.** (1995). Life history data, organochlorine pollutants and parasites from eight Burmeister’s porpoises, Phocoena spinipinnis, caught in northern Argentine waters. *Report of the International Whaling Commission*.

**Cowan, D. F.** (1967). Helminth Parasites of the Pilot Whale Globicephala melaena (Traill 1809). *The Journal of Parasitology* **53**, 166. doi: 10.2307/3276641.

**Dagleish, M. P., Barley, J., Finlayson, J., Reid, R. J. and Foster, G.** (2008). Brucella ceti Associated Pathology in the Testicle of a Harbour Porpoise (Phocoena phocoena). *Journal of Comparative Pathology* **139**, 54–59. doi: 10.1016/j.jcpa.2008.03.004.

**Dailey, M. D.** (1970). The Transmission of Parafilaroides decorus (Nematoda: Metastrongyloidea) in the California Sea Lion. *Proceedings of the Helminthological Society of Washington* **37**, 215–222.

**Dailey, M. D.** (2006). Restoration of Parafilaroides (Dougherty, 1946) (Nematoda: Metastrongyloidea) with Description of Two New Species from Pinnipeds of Eastern Central Pacific. *Journal of Parasitology* **92**, 589–594. doi: 10.1645/GE-3525.1.

**Dailey, M. D.** (2009). A New Species of Parafilaroides (Nematoda: Filaroididae) in Three Species of Fur Seals (Carnivora: Otariidae) From the Southern Hemisphere. *Journal of Parasitology* **95**, 156–159. doi: 10.1645/GE-1521.1.

**Dailey, M. D. and Brownell, R. L. Jr.** (1972). A checklist of marine mammal parasites. In *Mammals of the sea: Biology and medicine, SH Ridgway (ed.). Charles C Thomas, Springfield, Illinois*, pp. 528–589.

**Dailey, M. D. and Perrin, W. F.** (1973). Helminth parasities of porpoises of the genus Stenella in the eastern tropical pacific, with descriptions of two new species: Mastigonema stenellae gen. et. sp. n. (Nematoda: spiruroidea) and Zalophotrema pacificum sp. n. (Trematoda: digenea). *Fishery Bulletin* **71**, 455–471.

**Dailey, M. and Stroud, R.** (1978). Parasites and associated pathology observed in cetaceans stranded along the Oregon coast. *Journal of Wildlife Diseases* **14**, 503–511. doi: 10.7589/0090-3558-14.4.503.

**Dailey, M. D. and Walker, W. A.** (1978). Parasitism as a Factor (?) in Single Strandings of Southern California Cetaceans. *The Journal of Parasitology* **64**, 593. doi: 10.2307/3279939.

**Dailey, M., Walsh, M., Odell, D. and Campbell, T.** (1991). Evidence of prenatal infection in the bottlenose dolphin (Tursiops truncatus) with the lungworm Halocercus lagenorhynchi (Nematoda: Pseudaliidae). *Journal of Wildlife Diseases* **27**, 164–165. doi: 10.7589/0090-3558-27.1.164.

**Davison, N. J., Simpson, V. R., Chappell, S., Monies, R. J., Stubberfield, E. J., Koylass, M., Quinney, S., Deaville, R., Whatmore, A. M. and Jepson, P. D.** (2010a). Prevalence of a host-adapted group B Salmonella enterica in harbour porpoises (Phocoena phocoena) from the south-west coast of England. *Veterinary Record* **167**, 173–176. doi: 10.1136/vr.c3760.

**Davison, N., Barnett, J., Rule, B., Chappell, S. and Wise, G.** (2010b). Group B Salmonella in lungworms from a harbour porpoise (Phocoena phocoena). *Veterinary Record* **167**, 351–352. doi: 10.1136/vr.c4495.

**Davison, N. J., Barnett, J. E. F., Perrett, L. L., Dawson, C. E., Perkins, M. W., Deaville, R. C. and Jepson, P. D.** (2013). Meningoencephalitis and Arthritis Associated with Brucella ceti in a Short-beaked Common Dolphin (Delphinus delphis). *Journal of Wildlife Diseases* **49**, 632–636. doi: 10.7589/2012-06-165.

**Dawson, C. E., Perrett, L. L., Stubberfield, E. J., Stack, J. A., Farrelly, S. S. J., Cooley, W. A., Davison, N. J. and Quinney, S.** (2008). Isolation and characterization of Brucella from the lungworms of a Harbour porpoise (Phocoena phocoena). *Journal of Wildlife Diseases* **44**, 237–246. doi: 10.7589/0090-3558-44.2.237.

**de Castro, R. L., Vales, D. G., Degrati, M., García, N., Fernández, M. and Crespo, E. A.** (2014). First record of cestode cysts of Phyllobothrium delphini (Phyllobothriidae) from dusky dolphins (Lagenorhynchus obscurus) off Argentine coast. *Hidrobiológica* **24**, 307–310.

**Delyamure, S. L.** (1968). *Helminthofauna of Marine Mammals: Ecology and Phylogeny*. ed. Skri︠a︡bin, K. I. Israel Program for Scientific Translations.

**Delyamure, S., Yurakhno, M., Popov, V. N., Shults, L. M. and Fay, F. H.** (1984). Helminthological comparison of subpopulations of Bering Sea spotted seals, Phoca largha Pallas. *Soviet-American cooperative research on marine mammals* **1**, 61–65.

**Dennison, S., Gulland, F., Haulena, M., De Morais, H. and Colegrove, K.** (2007). Urate nephrolithiasis in a northern elephant seal (Mirounga angustirostris) and a California sea lion (Zalophus californianus). *Journal of Zoo and Wildlife Medicine* **38**, 114–120. doi: 10.1638/05-121.1.

**Di Beneditto, A. P. M. and Ramos, R. M. A.** (2004). Biology of the marine tucuxi dolphin (Sotalia fluviatilis) in south-eastern Brazil. *Journal of the Marine Biological Association of the United Kingdom* **84**, 1245–1250. doi: 10.1017/S0025315404010744h.

**DÍaz-Delgado, J., Arbelo, M., Sacchini, S., Quesada-Canales, Ó., Andrada, M., Rivero, M. and Fernández, A.** (2012). Pulmonary Angiomatosis and Hemangioma in Common Dolphins (*Delphinus delphis*) Stranded in Canary Islands. *Journal of Veterinary Medical Science* **74**, 1063–1066. doi: 10.1292/jvms.11-0573.

**Domiciano, I. G., Domit, C., Broadhurst, M. K., Koch, M. S. and Bracarense, A. P. F. R. L.** (2016). Assessing Disease and Mortality among Small Cetaceans Stranded at a World Heritage Site in Southern Brazil. *PLOS ONE* **11**, e0149295. doi: 10.1371/journal.pone.0149295.

**Dougherty, E. C.** (1943). Notes on the lungworms of porpoises and their occurrence on the California coast. *Proceedings of the Helminthological Society of Washington* **10**, 16–22.

**Dougherty, E. C.** (1944). The lungworms (Nematoda: Pseudalidae) of Odontoceti. Part I. *Parasitology* **36**, 80–94.

**Dougherty, E. C. and Herman, C. M.** (1947). New species of the genus Parafilaroides Dougherty, 1946 (Nematoda: Metastrongylidae) from sea-lions, with a list of the lungworms of the Pinnipedia. *Proceedings of the Helminthological Society of Washington* **14**, 77–87.

**Dougnac, C. and Fredes, F.** (2012). *Identificación de fauna endoparasitaria en cetáceos de Tierra del Fuego: Nuevos registros de helmintos para la zona y para odontocetos de la región.* Editorial Academica Espanola.

**Duignan, P. J.** (2000). Diseases of cetaceans and pinnipeds. In *Marine wildlife ; the Fabian Fay course for veterinarians ; proceedings 335, 4-8 September 2000*, pp. 419–447. Univ. of Sydney, Sydney.

**Dunn, J. L. and Wolke, R. E.** (1976). Dipetalonema spirocauda infection in the atlantic harbour seal (Phoca vitulina concolor). *Journal of Wildlife Diseases* **12**, 531–538. doi: 10.7589/0090-3558-12.4.531.

**Ebmer, D., Navarrete, M. J., Muñoz, P., Flores, L. M., Gärtner, U., Brabec, J., Poppert, S., Taubert, A. and Hermosilla, C.** (2020). Anthropozoonotic Parasites Circulating in Synanthropic and Pacific Colonies of South American Sea Lions (Otaria flavescens): Non-invasive Techniques Data and a Review of the Literature. *Frontiers in Marine Science* **7**, 543829. doi: 10.3389/fmars.2020.543829.

**Echenique, J., Pereira, E., Prado, J., Schild, A. L. and Valente, A. L.** (2020). New host and geographical records for Parafilaroides normani (Nematoda: Filaroididae) Dailey, 2009 in South American fur seal, Arctocephalus australis, from southern Brazil. *Journal of Helminthology* **94**, 1–4. doi: 10.1017/S0022149X20000826.

**Elson-Riggins, J. G., Al-Banna, L., Platzer, E. G. and Kaloshian, I.** (2001). Characterization of Otostrongylus circumlitis from pacific harbor and northern elephant seals. *Journal of Parasitology* **87**, 73–78.

**Elson-Riggins, J. G., Riggins, S. A., Gulland, F. M. D. and Platzer, E. G.** (2004). Immunoglobulin responses of northern elephant and Pacific harbor seals naturally infected with Otostrongylus circumlitus. *Journal of Wildlife Diseases* **40**, 466–475. doi: 10.7589/0090-3558-40.3.466.

**Elson-Riggins, J. G., Gibbons, L. M., Van Liere, D. W., Zinkstok, E. W., Blake, D. P., Alegre, F., Spittle, H., Brakefield, P. M., Udo de Haes, H. A. and Osinga, N.** (2020). Surprisingly long body length of the lungworm Parafilaroides gymnurus from common seals of the Dutch North Sea. *Parasitology Research* **119**, 1803–1817. doi: 10.1007/s00436-020-06675-7.

**Evans, P.** (1991). Whales, dolphins and porpoises: order Cetacea. In *The handbook of British mammals*, pp. 299–350. Blackwell Oxford.

**Evans, R. H.** (2011). Segniliparus rugosus–associated Bronchiolitis in California Sea Lion. *Emerging Infectious Diseases* **17**, 311–312. doi: 10.3201/eid1702.101511.

**Ewing, R. Y., Rotstein, D. S., McLellan, W. A., Costidis, A. M., Lovewell, G., Schaefer, A. M., Romero, C. H. and Bossart, G. D.** (2020). Macroscopic and Histopathologic Findings From a Mass Stranding of Rough-Toothed Dolphins (Steno bredanensis) in 2005 on Marathon Key, Florida, USA. *Frontiers in Veterinary Science* **7**, 572. doi: 10.3389/fvets.2020.00572.

**Faulkner, J.** (1995). Study of the cranial sinus nematode Stenurus minor, Metastrongyloidea, in the harbour porpoise, Phocoena phocoena.

**Faulkner, J., Measures, L. N. and Whoriskey, F. G.** (1998). Stenurus minor (Metastrongyloidea: Pseudaliidae) infections of the cranial sinuses of the harbour porpoise, Phocoena phocoena. *Canadian Journal of Zoology* **76**, 1209–1216.

**Fauquier, D., Gulland, F., Haulena, M. and Spraker, T.** (2003). Biliary Adenocarcinoma in a Stranded Northern Elephant Seal (Mirounga angustirostris). *Journal of Wildlife Diseases* **39**, 723–726. doi: 10.7589/0090-3558-39.3.723.

**Fauquier, D., Gulland, F., Haulena, M., Dailey, M., Rietcheck, R. L. and Lipscomb, T. P.** (2004). Meningoencephalitis in Two Stranded California Sea Lions (Zalophus californianus) Caused by Aberrant Trematode Migration. *Journal of Wildlife Diseases* **40**, 816–819. doi: 10.7589/0090-3558-40.4.816.

**Fauquier, D., Kinsel, M., Dailey, M., Sutton, G., Stolen, M., Wells, R. and Gulland, F.** (2009). Prevalence and pathology of lungworm infection in bottlenose dolphins Tursiops truncatus from southwest Florida. *Diseases of Aquatic Organisms* **88**, 85–90. doi: 10.3354/dao02095.

**Fenton, H., Daoust, P., Forzán, M., Vanderstichel, R., Ford, J., Spaven, L., Lair, S. and Raverty, S.** (2017). Causes of mortality of harbor porpoises Phocoena phocoena along the Atlantic and Pacific coasts of Canada. *Diseases of Aquatic Organisms* **122**, 171–183. doi: 10.3354/dao03080.

**Fernández, M., Agustí, C., Aznar, F. and Raga, J.** (2003). Gastrointestinal helminths of Risso’s dolphin Grampus griseus from the Western Mediterranean. *Diseases of Aquatic Organisms* **55**, 73–76. doi: 10.3354/dao055073.

**Fleischman, R. W. and Squire, R. A.** (1970). Verminous Pneumonia in the California Sea Lion (Zalophus californianus). *Pathologia veterinaria* **7**, 89–101. doi: 10.1177/030098587000700201.

**Forrester, D. J., Odell, D. K., Thompson, N. P. and White, J. R.** (1980). Morphometrics, Parasites, and Chlorinated Hydrocarbon Residues of Pygmy Killer Whales from Florida. *Journal of Mammalogy* **61**, 356–360. doi: 10.2307/1380067.

**Fouchier, R. A. M., Bestebroer, T. M., Martina, B. E. E., Rimmelzwaan, G. F. and Osterhaus, A. D. M. E.** (2001). Infection of grey seals and harbour seals with influenza B virus. *International Congress Series* **1219**, 225–231. doi: 10.1016/S0531-5131(01)00647-1.

**Gabel, M., Theisen, S., Palm, H. W., Dähne, M. and Unger, P.** (2021). Nematode Parasites in Baltic Sea Mammals, Grey Seal (Halichoerus grypus (Fabricius, 1791)) and Harbour Porpoise (Phocoena phocoena (L.)), from the German Coast. *Acta Parasitologica* **66**, 26–33. doi: 10.1007/s11686-020-00246-7.

**García de los Ríos y Loshuertos, A., Soler Laguía, M., Arencibia Espinosa, A., López Fernández, A., Covelo Figueiredo, P., Martínez Gomariz, F., Sánchez Collado, C., García Carrillo, N. and Ramírez Zarzosa, G.** (2021). Comparative Anatomy of the Nasal Cavity in the Common Dolphin Delphinus delphis L., Striped Dolphin Stenella coeruleoalba M. and Pilot Whale Globicephala melas T.: A Developmental Study. *Animals* **11**, 441. doi: 10.3390/ani11020441.

**Garner, M. M., Lambourn, D. M., Jeffries, S. J., Hall, P. B., Rhyan, J. C., Ewalt, D. R., Polzin, L. M. and Cheville, N. F.** (1997). Evidence of Brucella Infection in Parafilaroides Lungworms in a Pacific Harbor Seal (Phoca Vitulina Richardsi). *Journal of Veterinary Diagnostic Investigation* **9**, 298–303. doi: 10.1177/104063879700900311.

**Gerber, J. A., Roletto, J., Morgan, L. E., Smith, D. M. and Gage, L. J.** (1993). Findings in pinnipeds stranded along the central and northern California coast, 1984–1990. *Journal of Wildlife Diseases* **29**, 423–433. doi: 10.7589/0090-3558-29.3.423.

**Gibson, D. I. and Harris, E. A.** (1979). The helminth-parasites of cetaceans in the collection of the British Museum (Natural History). *Investigations on Cetacea* **10**, 309–324.

**Gibson, D. I., Harris, E. A., Bray, R. A., Jepson, P. D., Kuiken, T., Baker, J. R. and Simpson, V. R.** (1998). A survey of the helminth parasites of cetaceans stranded on the coast of England and Wales during the period 1990-1994. *Journal of Zoology* **244**, 563–574. doi: 10.1111/j.1469-7998.1998.tb00061.x.

**Goldstein, T., Colegrove, K., Hanson, M. and Gulland, F.** (2011). Isolation of a novel adenovirus from California sea lions Zalophus californianus. *Diseases of Aquatic Organisms* **94**, 243–248. doi: 10.3354/dao02321.

**Goodall, R., Galeazzi, A., Leatherwood, S., Miller, K., Cameron, I., Kastelein, R. and Sobral, A.** (1988). Studies of Commerson’s dolphins, Cephalorhynchus commersonii, off Tierra del Fuego, 1976-1984, with a review of information on the species in the South Atlantic. *Report of the International Whaling Commission* 3–70.

**Gosselin, J.-F. and Measures, L. N.** (1997). Redescription of Filaroides (Parafilaroides) gymnurus( Railliet, 1899) (Nematoda: Metastrongyloidea), with comments on other species in pinnipeds. *Canadian Journal of Zoology* **75**, 359–370. doi: 10.1139/z97-045.

**Gosselin, J.-F., Measures, L. N. and Huot, J.** (1998). Lungworm (Nematoda: Metastrongyloidea) infections in Canadian phocids. *Canadian Journal of Fisheries and Aquatic Sciences* **55**, 825–834. doi: 10.1139/f97-306.

**Greenwood, A. G. and Taylor, D. C.** (1979). Odontocete parasites: Some new host records. *Aquatic Mammals* **7**, 23–25.

**Greig, D. J., Gulland, F. M. D. and Kreuder, C.** (2005). A Decade of Live California Sea Lion (Zalophus californianus) Strandings Along the Central California Coast: Causes and Trends, 1991-2000. *Aquatic Mammals* **31**, 11–22. doi: 10.1578/AM.31.1.2005.11.

**Greig, D., Gulland, F., Smith, W., Conrad, P., Field, C., Fleetwood, M., Harvey, J., Ip, H., Jang, S., Packham, A., Wheeler, E. and Hall, A.** (2014). Surveillance for zoonotic and selected pathogens in harbor seals Phoca vitulina from central California. *Diseases of Aquatic Organisms* **111**, 93–106. doi: 10.3354/dao02762.

**Groch, K. R., Santos-Neto, E. B., Díaz-Delgado, J., Ikeda, J. M. P., Carvalho, R. R., Oliveira, R. B., Guari, E. B., Bisi, T. L., Azevedo, A. F., Lailson-Brito, J. and Catão-Dias, J. L.** (2018). Guiana Dolphin Unusual Mortality Event and Link to Cetacean Morbillivirus, Brazil. *Emerging Infectious Diseases* **24**, 1349–1354. doi: 10.3201/eid2407.180139.

**Groch, K. R., Díaz-Delgado, J., Santos-Neto, E. B., Ikeda, J. M. P., Carvalho, R. R., Oliveira, R. B., Guari, E. B., Flach, L., Sierra, E., Godinho, A. I., Fernández, A., Keid, L. B., Soares, R. M., Kanamura, C. T., Favero, C., Ferreira-Machado, E., Sacristán, C., Porter, B. F., Bisi, T. L., Azevedo, A. F., Lailson-Brito, J. and Catão-Dias, J. L.** (2020). The Pathology of Cetacean Morbillivirus Infection and Comorbidities in Guiana Dolphins During an Unusual Mortality Event (Brazil, 2017–2018). *Veterinary Pathology* **57**, 845–857. doi: 10.1177/0300985820954550.

**Gui, D., He, J., Zhang, X., Tu, Q., Chen, L., Feng, K., Liu, W., Mai, B. and Wu, Y.** (2018). Potential association between exposure to legacy persistent organic pollutants and parasitic body burdens in Indo-Pacific finless porpoises from the Pearl River Estuary, China. *Science of The Total Environment* **643**, 785–792. doi: 10.1016/j.scitotenv.2018.06.249.

**Guimarães, J. P., Batista, R. L. G., Mariani, D. B. and Vergara-Parente, J. E.** (2013). Ingestion of plastic debris by estuarine dolphin, Sotalia guianensis, off northeastern Brazil. *Arquivos de Ciências do Mar* **46**, 107–122.

**Guimarães, J. P., Febronio, A. M. B., Vergara-Parente, J. E. and Werneck, M. R.** (2015). Lesions Associated with Halocercus brasiliensis Lins de Almeida, 1933 in the Lungs of Dolphins Stranded in the Northeast of Brazil. *Journal of Parasitology* **101**, 248–251. doi: 10.1645/14-513.1.

**Gulland, F. M. D., Beckmen, K., Burek, K., Lowenstine, L., Werner, L., Spraker, T., Dailey, M. and Harris, E.** (1997). Nematode (Otostrongylus circumlitus) infestation of northern elephant seals (Mirounga angustirostris) stranded along the central California coast. *Marine Mammal Science* **13**, 446–458. doi: 10.1111/j.1748-7692.1997.tb00651.x.

**Gulland, F. M. D., Hall, A. J., Greig, D. J., Frame, E. R., Colegrove, K. M., Booth, R. K. N., Wasser, S. K. and Scott-Moncrieff, J. C. R.** (2012). Evaluation of circulating eosinophil count and adrenal gland function in California sea lions naturally exposed to domoic acid. *Journal of the American Veterinary Medical Association* **241**, 943–949. doi: 10.2460/javma.241.7.943.

**Harris, E.** (1982). The helminth parasites of the Cetacea (or Parasitology with a porpoise).pp. R71–R71. Cambridge Univ Press.

**Haulena, M. and Gulland, F. M. D.** (2001). Use of medetomidine-zolazepam-tiletamine with and without atipamezole reversal to immobilize captive california sea lions. *Journal of Wildlife Diseases* **37**, 566–573. doi: 10.7589/0090-3558-37.3.566.

**Hermosilla, C., Silva, L. M. R., Navarro, M. and Taubert, A.** (2016). Anthropozoonotic Endoparasites in Free-Ranging “Urban” South American Sea Lions ( *Otaria flavescens* ). *Journal of Veterinary Medicine* **2016**, 1–7. doi: 10.1155/2016/7507145.

**Hernández-Orts, J. S., Hernández-Mena, D. I., Pantoja, C., Kuchta, R., García, N. A., Crespo, E. A. and Loizaga, R.** (2021). A Visitor of Tropical Waters: First Record of a Clymene Dolphin (Stenella clymene) Off the Patagonian Coast of Argentina, With Comments on Diet and Metazoan Parasites. *Frontiers in Marine Science* **8**, 658975. doi: 10.3389/fmars.2021.658975.

**Herreman, J. K., McIntosh, A. D., Dziuba, R. K., Blundell, G. M., Ben-David, M. and Greiner, E. C.** (2011). Parasites of harbor seals (Phoca vitulina) in Glacier Bay and Prince William Sound, Alaska. *Marine Mammal Science* **27**, 247–253. doi: 10.1111/j.1748-7692.2009.00355.x.

**Houde, M., Measures, L. N. and Huot, J.** (2003). Lungworm (Pharurus pallasii: Metastrongyloidea: Pseudaliidae) infection in the endangered St. Lawrence beluga whale (Delphinapterus leucas). *Canadian Journal of Zoology* **81**, 543–551.

**Hsü, H. and Hoeppli, R.** (1933). On some parasitic nematodes collected in Amoy. *Peking Natural History Bulletin* **8**, 155–168.

**Huertas, V. and Lagueux, C. J.** (2016). First Recorded Mass Stranding of the Short-Finned Pilot Whale (Globicephala macrorhynchus) on the Caribbean Coast of Nicaragua. *Aquatic Mammals* **42**, 27–34. doi: 10.1578/AM.42.1.2016.27.

**Jabbar, A., Mohandas, N. and Gasser, R. B.** (2014). Characterisation of the mitochondrial genome of Parafilaroides normani (lungworm) of Arctocephalus pusillus doriferus (Australian fur seal). *Parasitology Research* **113**, 3049–3055. doi: 10.1007/s00436-014-3968-8.

**Jacobus, K., Marigo, J., Gastal, S. B., Taniwaki, S. A., Ruoppolo, V., Catão-Dias, J. L. and Tseng, F.** (2016). Identification of respiratory and gastrointestinal parasites of three species of pinnipeds Arctocephalus australis, Arctocephalus gazella, and Otaria flavescens in southern Brazil. *Journal of Zoo and Wildlife Medicine* **47**, 132–140. doi: 10.1638/2015-0090.1.

**Jauniaux, T., Petitjean, D., Brenez, C., Borrens, M., Brosens, L., Haelters, J., Tavernier, T. and Coignoul, F.** (2002). Post-mortem Findings and Causes of Death of Harbour Porpoises (Phocoena phocoena) Stranded from 1990 to 2000 along the Coastlines of Belgium and Northern France. *Journal of Comparative Pathology* **126**, 243–253. doi: 10.1053/jcpa.2001.0547.

**Jepson, P. D., Kuiken, T., Bennett, P. M., Baker, J. R., Simpson, V. R. and Kennedy, S.** (2000). Pulmonary pathology of harbour porpoises (Phocoena phocoena) stranded in England and Wales between 1990 and 1996. *Veterinary Record* **146**, 721–728. doi: 10.1136/vr.146.25.721.

**Johnston, D. G. and Ridgway, S. H.** (1969). Parasitism in some marine mammals. *Journal of the American Veterinary Medical Association* **155**, 1064–1072.

**Kastelein, R. A. and Lavaleije, M. S. S.** (1992). Foreign Bodies in the stomach of female Habrour porpoises (Phocoena phocoena) from the North Sea. *Aquatic Mammals* **18**, 40–46.

**Kaye, S., Johnson, S., Arnold, R. D., Nie, B., Davis, J. T., Gulland, F., Abou-Madi, N. and Fletcher, D. J.** (2016). Pharamcokinetic study of oral e-aminocaproic acid in the northern elephant seal (Mirounga Angustirostris). *Journal of Zoo and Wildlife Medicine* **47**, 438–446. doi: 10.1638/2015-0138.1.

**Kaye, S., Johnson, S., Rios, C. and Fletcher, D. J.** (2017). Plasmatic coagulation and fibrinolysis in healthy and Otostrongylus -affected Northern elephant seals (Mirounga angustirostris). *Veterinary Clinical Pathology* **46**, 589–596. doi: 10.1111/vcp.12540.

**Kelly, T. R., Greig, D., Colegrove, K. M., Lowenstine, L. J., Dailey, M., Gulland, F. M. and Haulena, M.** (2005). Metastrongyloid Nematode (Otostrongylus circumlitus) Infection in a Stranded California Sea Lion (Zalophus californianus)— a New Host-parasite Association. *Journal of Wildlife Diseases* **41**, 593–598. doi: 10.7589/0090-3558-41.3.593.

**Kennedy, M. J.** (1986). Metastrongyloidea) from the lungs of the ringed seal, Phoca hispida (Phocidae), from the Beaufort Sea, Canada. *Canadian Journal of Zoology* **64**, 1864–1868. doi: 10.1139/z86-278.

**Kennedy, S., Smyth, J. A., Cush, P. F., Duignan, P., Platten, M., McCullough, S. J. and Allan, G. M.** (1989). Histopathologic and Immunocytochemical Studies of Distemper in Seals. *Veterinary Pathology* **26**, 97–103. doi: 10.1177/030098588902600201.

**Kenyon, A. J. and Kenyon, B. J.** (1977). Prevalence of Pharurus pallasii in the beluga whale (Delphinapterus leucas) of Churchill River Basin, Manitoba. *Journal of Wildlife Diseases* **13**, 338–340. doi: 10.7589/0090-3558-13.4.338.

**Kijewska, A. P., Jankowski, Z., Kuklik, I. and Rokicki, J.** (2003). Pathological changes in the auditory organs of the harbor porpoise (Phocoena phocoena, L.) associated with Stenurus minor (Kuhn, 1829). *Acta Parasitologica* **48**, 60–63. doi: 10.13140/RG.2.1.4781.7046.

**Kontrimavichus, V. and Delyamure, S.** (1979). Filaroides of domestic and wild animals. *Fundamentals of nematology* **29**, 30–36.

**Kroese, M. V., Beckers, L., Bisselink, Y. J. W. M., Brasseur, S., van Tulden, P. W., Koene, M. G. J., Roest, H. I. J., Ruuls, R. C., Backer, J. A., IJzer, J., van der Giessen, J. W. B. and Willemsen, P. T. J.** (2018). Brucella pinnipedialis in grey seals (Halichoerus grypus) and harbour seals (Phoca vitulina) in the Netherlands. *Journal of Wildlife Diseases* **54**, 439. doi: 10.7589/2017-05-097.

**Kühn, J.** (1829). Description d’un nouvelle espèce de strongyle trouveé dans le marsouin. *Bulletin du Sciences Naturelles et Geologie* **17**, 150–153.

**Kumazawa, H., Yona, R., Hirai, M. and Hasegawa, H.** (2006). A Fatal Case of Bronchopneumonia Associated with Lungworm lnfection in a Bottle-nosed dolphin, tursiops truncatus (Cetacea: Dephinidae). *Japanese Journal of Zoo and Wildlife Medicine* **11**, 31–34.

**Kuramochi, T., Araki, J. and Machida, M.** (1990). Pseudaliid nematodes from Dall’s porpoise, Phocoenoides dalli. *Bulletin of the National Science Museum Series A (Zoology* 97–103.

**Kuramochi, T., Kikuchi, T., Okamura, H., Tatsukawa, T., Doi, H., Nakamura, K., Yamada, T., Koda, Y., Yoshida, Y. and Matsuura, M.** (2000). Parasitic helminth and epizoit fauna of finless porpoise in the Inland Sea of Japan and the western North Pacific with a preliminary note on faunal difference by host’s local population. *Memoirs of the National Science Museum, Tokyo* **33**, 83–95.

**Kurochkin, Y. V. and Zablotsky, V.** (1958). On the helminth fauna of the Caspian seal [In Russian]. *Trudy Astrakhan—skogo Zapovednika 1993* 337–343.

**Kuwamura, M., Sawamoto, O., Yamate, J., Aoki, M., Ohnishi, Y. and Kotani, T.** (2007). Pulmonary Vascular Proliferation and Lungworm (Stenurus ovatus) in a Bottlenose Dolphin (Tursiops turncatus). *Journal of Veterinary Medical Science* **69**, 531–533. doi: 10.1292/jvms.69.531.

**Kuzmina, T. A., Spraker, T. R., Kudlai, O., Lisitsyna, O. I., Zabludovskaja, S. O., Karbowiak, G., Fontaine, C. and Kuchta, R.** (2018). Metazoan parasites of California sea lions (Zalophus californianus): A new data and review. *International Journal for Parasitology: Parasites and Wildlife* **7**, 326–334. doi: 10.1016/j.ijppaw.2018.09.001.

**Lambourn, D. M., Garner, M., Ewalt, D., Raverty, S., Sidor, I., Jeffries, S. J., Rhyan, J. and Gaydos, J. K.** (2013). Brucella pinnipedialis infections in pacific harbour seals Phoca vitulina richardsi from Washington State, USA. *Journal of Wildlife Diseases* **49**, 802–815. doi: 10.7589/2012-05-137.

**Lane, E. P., de Wet, M., Thompson, P., Siebert, U., Wohlsein, P. and Plön, S.** (2014). A Systematic Health Assessment of Indian Ocean Bottlenose (Tursiops aduncus) and Indo-Pacific Humpback (Sousa plumbea) Dolphins Incidentally Caught in Shark Nets off the KwaZulu-Natal Coast, South Africa. *PLoS ONE* **9**,. doi: 10.1371/journal.pone.0107038.

**Lehnert, K., Raga, J. and Siebert, U.** (2005). Macroparasites in stranded and bycaught harbour porpoises from German and Norwegian waters. *Diseases of Aquatic Organisms* **64**, 265–269. doi: 10.3354/dao064265.

**Lehnert, K., Raga, J. A. and Siebert, U.** (2007). Parasites in harbour seals (Phoca vitulina) from the German Wadden Sea between two Phocine Distemper Virus epidemics. *Helgoland Marine Research* **61**, 239–245. doi: 10.1007/s10152-007-0072-9.

**Lehnert, K., von Samson-Himmelstjerna, G., Schaudien, D., Bleidorn, C., Wohlsein, P. and Siebert, U.** (2010). Transmission of lungworms of harbour porpoises and harbour seals: Molecular tools determine potential vertebrate intermediate hosts. *International Journal for Parasitology* **40**, 845–853. doi: 10.1016/j.ijpara.2009.12.008.

**Lehnert, K., Seibel, H., Hasselmeier, I., Wohlsein, P., Iversen, M., Nielsen, N. H., Heide-Jørgensen, M. P., Prenger-Berninghoff, E. and Siebert, U.** (2014). Increase in parasite burden and associated pathology in harbour porpoises (Phocoena phocoena) in West Greenland. *Polar Biology* **37**, 321–331. doi: 10.1007/s00300-013-1433-2.

**Lehnert, K., Randhawa, H. and Poulin, R.** (2017). Metazoan parasites from odontocetes off New Zealand: new records. *Parasitology Research* **116**, 2861–2868. doi: 10.1007/s00436-017-5573-0.

**Leidenberger, S. and Boström, S.** (2009). Description of the lungworm Otostrongylus circumlitus (Railliet, 1899) de Bruyn, 1933 (Metastrongyloidea: Crenosomatidae) found in the heart of harbour seals from Sweden. *Journal of Nematode Morphology and Systematics* **12**, 169–175.

**Lipscomb, T. P., Kennedy, S., Moffett, D., Krafft, A., Klaunberg, B. A., Lichy, J. H., Regan, G. T., Worthy, G. A. J. and Taubenberger, J. K.** (1996). Morbilliviral Epizootic in Bottlenose Dolphins of the Gulf of Mexico. *Journal of Veterinary Diagnostic Investigation* **8**, 283–290. doi: 10.1177/104063879600800302.

**Liu, H., Plancarte, M., Ball, E. E., Weiss, C. M., Gonzales-Viera, O., Holcomb, K., Ma, Z.-M., Allen, A. M., Reader, J. R., Duignan, P. J., Halaska, B., Khan, Z., Kriti, D., Dutta, J., van Bakel, H., Jackson, K., Pesavento, P. A., Boyce, W. M. and Coffey, L. L.** (2021). Respiratory Tract Explant Infection Dynamics of Influenza A Virus in California Sea Lions, Northern Elephant Seals, and Rhesus Macaques. *Journal of Virology* **95**,. doi: 10.1128/JVI.00403-21.

**Lucas, Z., Daoust, P.-Y., Conboy, G. and Brimacombe, M.** (2003). Health status of harp seals (Phoca groenlandica) and hooded seals (Cystophora cristata) on Sable Island, Nova Scotia, Canada, concurrent with their expanding range. *Journal of Wildlife Diseases* **39**, 16–28. doi: 10.7589/0090-3558-39.1.16.

**MacNeill, A. C., Neufeld, J. L. and Webster, W. A.** (1975). Pulmonary nematodiasis in a narwhale. *The Canadian Veterinary Journal = La Revue Veterinaire Canadienne* **16**, 53–55.

**Marigo, J., Ruoppolo, V., Rosas, F. C. W., Valente, A. L. S., Oliveira, M. R., Dias, R. A. and Catão-Dias, J. L.** (2010). Helminths of Sotalia guianensis (Cetacea: Delphinidae) from the South and Southeastern Coasts of Brazil. *Journal of Wildlife Diseases* **46**, 599–602. doi: 10.7589/0090-3558-46.2.599.

**Mawson, P. M.** (1953). Parasitic Nematoda collected by the Australian National Antarctic Research Expedition: Heard Island and Macquarie Island, 1948–1951. *Parasitology* **43**, 291–297. doi: 10.1017/S0031182000018667.

**Mazzariol, S., Marruchella, G., Di Guardo, G., Podesta, M., Olivieri, V., Colangelo, P., Kennedy, S., Castagnaro, M. and Cozzi, B.** (2007). Post-mortem Findings in Cetacean Stranded along Italian Adriatic Sea coastline (2000-2006).

**McColl, K. A. and Obendorf, D. L.** (1982). Helminth parasites and associated pathology in stranded frasers dolphins, Lagenodelohis Hosei (Fraser, 1956). *Aquatic Mammals* **9**, 30–34.

**McFee, W. and Lipscomb, T. P.** (2009). Major pathological findings and probable causes of mortality in bottlenose dolphins stranded in South Carolina from 1993 to 2006. *Journal of Wildlife Diseases* **45**, 575–593. doi: 10.7589/0090-3558-45.3.575.

**McKenzie, J. and Blair, D.** (1983). Parasites from Hector’s dolphin (Cephalorhynchus hectori).pp. 126–127. Sir Publishing po box 399, Wellington, New zealand.

**McKnight, C. A., Reynolds, T. L., Haulena, M., deLahunta, A. and Gulland, F. M. D.** (2005). Congenital Hemicerebral Anomaly in a Stranded Pacific Harbor Seal (Phoca vitulina richardsi). *Journal of Wildlife Diseases* **41**, 654–658. doi: 10.7589/0090-3558-41.3.654.

**McManus, T., Wapstra, J., Guiler, E., Munday, B. and Obendorf, D.** (1984). Cetacean strandings in Tasmania from February 1978 to May 1983. *Papers and Proceedings of The Royal Society of Tasmania* **118**, 117–135. doi: 10.26749/rstpp.118.117.

**Measures, L. N. and Gosselin, J.** (1994). Helminth parasites of ringed seal, Phoca hispida, from northern Quebec, Canada. *Journal of the Helminthological Society of Washington* **61**, 240–244.

**Measures, L. N., Béland, P., Martineau, D. and Guise, S. D.** (1995). Helminths of an endangered population of belugas, Delphinapterus leucas , in the St. Lawrence estuary, Canada. *Canadian Journal of Zoology* **73**, 1402–1409. doi: 10.1139/z95-165.

**Melo, O. P., Ramos, R. M. A. and Di Beneditto, A. P. M.** (2006). Helminths of the marine tucuxi, Sotalia fluviatilis (Gervais, 1853) (Cetacea: Delphinidae), in northern Rio de Janeiro State, Brazil. *Brazilian Archives of Biology and Technology* **49**, 145–148. doi: 10.1590/S1516-89132006000100017.

**Migaki, G., Van Dyke, D. and Hubbard, R. C.** (1971). Some histopathological lesions caused by helminths in marine mammals. *Journal of Wildlife Diseases* **7**, 281–289.

**Mignucci-Giannoni, A. A., Rodríguez-López, M. A., Perez-Zayas, J. J., Montoya-Ospina, R. A. and Williams, E. H. J.** (1998a). First record of the melonhead whale for Puerto Rico. *Mammalia* **62**, 452–457.

**Mignucci-Giannoni, A. A., Hoberg, E. P., Siegel-Causey, D. and Williams, E. H.** (1998b). Metazoan Parasites and Other Symbionts of Cetaceans in the Caribbean. *The Journal of Parasitology* **84**, 939. doi: 10.2307/3284625.

**Morales Vela, B. and Olvera Gómez, L. D.** (1993). Varamiento de calderones Globicephala macrorhynchus (Cetacea: Delphinidae) en la Isla de Cozumel, Quintana Roo, México. *Anales del Instituto de Biología serie Zoología* **64**,.

**Morell, M., Lehnert, K., IJsseldijk, L., Raverty, S., Wohlsein, P., Gröne, A., André, M., Siebert, U. and Shadwick, R.** (2017). Parasites in the inner ear of harbour porpoise: cases from the North and Baltic Seas. *Diseases of Aquatic Organisms* **127**, 57–63. doi: 10.3354/dao03178.

**Moser, M. and Rhinehart, H.** (1993). The lungworm, Halocercus spp. (Nematoda: Pseudaliidae) in cetaceans from California. *Journal of Wildlife Diseases* **29**, 507–508. doi: 10.7589/0090-3558-29.3.507.

**Nicholson, A. and Fanning, J.** (1981). Parasites and associated pathology of the respiratory tract of the Australian sea lion: Neophoca cinerea.pp. 178–181.

**Oliveira, J. B., Morales, J. A., González-Barrientos, R. C., Hernández-Gamboa, J. and Hernández-Mora, G.** (2011). Parasites of cetaceans stranded on the Pacific coast of Costa Rica. *Veterinary Parasitology* **182**, 319–328. doi: 10.1016/j.vetpar.2011.05.014.

**Onderka, D. K.** (1989). Prevance and pathology of nematode infections in the lungs of ringed seals (Phoca hispida) of the Western Arctic of Canada. *Journal of Wildlife Diseases* **25**, 218–224. doi: 10.7589/0090-3558-25.2.218.

**Osinga, N., Kappe, A. L., Brakefield, P. M., Udo de Haes, H. A. and Elson-Riggins, J. G.** (2015). Comparative biology of common and grey seals along the Dutch coast: stranding, disease, rehabilitation and conservation. Chapter 8: Observations regarding transmission of seal nematodes in common seals, Phoca vitulina vitulina from the Wadden Sea.

**Parsons, E. C. M. and Jefferson, T. A.** (2000). Post-mortem investigations on stranded dolphins and porpoises from Hong Kong waters. *Journal of Wildlife Diseases* **36**, 342–356. doi: 10.7589/0090-3558-36.2.342.

**Parsons, E. C. M., Overstreet, R. M. and Jefferson, T. A.** (2001). Parasites from Indo-Pacific hump-backed dolphins (Sousa chinensis) and finless porpoises (Neophocaena phocaenoides) stranded in Hong Kong. *Veterinary Record* **148**, 776–780. doi: 10.1136/vr.148.25.776.

**Pekmezci̇, G. Z., Yardimci, B., Gürler, A. T., Bölükbaş, C. S., Açici, M. and Umur, Ş.** (2013). Survey on the Presence of Nematodes and Associated with Pathology in Marine Mammals from Turkish Waters. *Kafkas Universitesi Veteriner Fakultesi Dergisi* **19**, 1035–1038. doi: 10.9775/kvfd.2013.9409.

**Perrin, W. F., Mitchell, E. D., Mead, J. G., Caldwell, D. K., Caldwell, M. C., Bree, P. J. H. and Dawbin, W. H.** (1987). Revision of the spotted dolphins, Stenella spp. *Marine Mammal Science* **3**, 99–170. doi: 10.1111/j.1748-7692.1987.tb00158.x.

**Perrin, W. F., Caldwell, D. K. and Caldwell, M.** (1994). Atlantic spotted dolphin Stenella frontalis (G. Cuvier, 1829). *Handbook of marine mammals* **5**, 173–190.

**Petter, A. J. and Pilleri, G.** (1982). Pharurus asiaeorientalis new species, metastrongylid nematode, parasite of Neophocaena asiaeorientalis (Phocoenidae, Cetacea). *Investigations on Cetacea* **13**, 141–148.

**Piché, C., Measures, L., Bédard, C. and Lair, S.** (2010). Bronchoalveolar lavage and pulmonary histopathology in harp seals (Phoca groenlandica) experimentally infected with Otostrongylus circumlitis. *Journal of Wildlife Diseases* **46**, 409–421. doi: 10.7589/0090-3558-46.2.409.

**Pool, R., Chandradeva, N., Gkafas, G., Raga, J. A., Fernández, M. and Aznar, F. J.** (2020a). Transmission and Predictors of Burden of Lungworms of the Striped Dolphin (Stenella coeruleoalba) in the Western Mediterranean. *Journal of Wildlife Diseases* **56**, 186. doi: 10.7589/2018-10-242.

**Pool, R., Fernández, M., Chandradeva, N., Raga, J. A. and Aznar, F. J.** (2020b). The taxonomic status of Skrjabinalius guevarai Gallego & Selva, 1979 (Nematoda: Pseudaliidae) and the synonymy of Skrjabinalius Delyamure, 1942 and Halocercus Baylis & Daubney, 1925. *Systematic Parasitology* **97**, 389–401. doi: 10.1007/s11230-020-09921-9.

**Pool, R., Romero-Rubira, C., Raga, J. A., Fernández, M. and Aznar, F. J.** (2021). Determinants of lungworm specificity in five cetacean species in the western Mediterranean. *Parasites & Vectors* **14**, 196. doi: 10.1186/s13071-021-04629-1.

**Prahl, S., Ketten, D. R. and Siebert, U.** (2008). Examinations of ears in Harbour porpoises Phocoena phocoena from the north and baltic seas. *Bioacoustics* **17**, 85–87. doi: 10.1080/09524622.2008.9753775.

**Prenger-Berninghoff, E., Siebert, U., Stede, M., König, A., Weiß, R. and Baljer, G.** (2008). Incidence of Brucella species in marine mammals of the German North Sea. *Diseases of Aquatic Organisms* **81**, 65–71. doi: 10.3354/dao01920.

**Raga, J. A.** (1994). Parasitismus bei den Cetacea. In *Robineau D, Duguy R, Klima M (eds.). Handbuch der Säugetiere Europas*, pp. 132–179.

**Raga, J. and Balbuena, J.** (1987). Algunas características zoogeográficas de los helmintos de los cetáceos en el Mediterráneo con especial referencia a la helmintofauna del delfín listado. *Mamíferos y helmintos. Barcelona: Ketres Editora* **195**, 201.

**Reckendorf, A., Ludes-Wehrmeister, E., Wohlsein, P., Tiedemann, R., Siebert, U. and Lehnert, K.** (2018). First record of Halocercus sp. (Pseudaliidae) lungworm infections in two stranded neonatal orcas (Orcinus orca). *Parasitology* **145**, 1553–1557. doi: 10.1017/S0031182018000586.

**Reisfeld, L., Sacristán, C., Sánchez-Sarmiento, A. M., Costa-Silva, S., Díaz-Delgado, J., Groch, K. R., Marigo, J., Ewbank, A. C., Favero, C. M., Guerra, J. M., Réssio, R. A., Cremer, M. J., Esperón, F. and Catão-Dias, J. L.** (2019). Fatal pulmonary parafilaroidiasis in a free-ranging subantarctic fur seal (Arctocephalus tropicalis) coinfected with two gammaherpesviruses and Sarcocystis sp. *Revista Brasileira de Parasitologia Veterinária* **28**, 499–503. doi: 10.1590/s1984-29612019029.

**Reyes, J. C. and van Waerebeek, K.** (1995). Aspects of the biology of Burmeister’s porpoise from Peru. *Report of the International Whaling Commission* 349–364.

**Rhyan, J., Garner, M., Spraker, T., Lambourn, D. and Cheville, N.** (2018). Brucella pinnipedialis in lungworms Parafilaroides sp. and Pacific harbor seals Phoca vitulina richardsi: proposed pathogenesis. *Diseases of Aquatic Organisms* **131**, 87–94. doi: 10.3354/dao03291.

**Rodrigues, T., Díaz-Delgado, J., Catão-Dias, J., da Luz Carvalho, J. and Marmontel, M.** (2018). Retrospective pathological survey of pulmonary disease in free-ranging Amazon river dolphin Inia geoffrensis and tucuxi Sotalia fluviatilis. *Diseases of Aquatic Organisms* **131**, 1–11. doi: 10.3354/dao03280.

**Rogan, E., Baker, J. R., Jepson, P. D., Berrow, S. and Kiely, O.** (1997). A mass stranding of white-sided dolphins (Lagenorhynchus acutus) in Ireland: biological and pathological studies. *Journal of Zoology* **242**, 217–227. doi: 10.1111/j.1469-7998.1997.tb05798.x.

**Rogan, E., Penrose, R., Gassner, I., Mackey, M. J. and Clayton, P.** (2001). *Marine Mammal Strandings: A Collaborative Study for the Irish Sea*. The Marine Institute.

**Rosas, F. C. W., Monteiro-Filho, E. L. A., Marigo, J., Santos, R. A., Andrade, A. L. V., Rautenberg, M., Olivcira, M. R. and Bordignon, M. O.** (2002). The striped dolphin, Stenella coeruleoalba (Cetacea: Delphinidae), on the coast of São Paulo State, southeastern Brazil. *Aquatic Mammals* **28**, 60–66.

**Ross, G. J. B. and Bass, A. J.** (1971). Shark attack on an ailing dolphin, Stenella Coeruleoalba (Meyen). *South African Journal of Science*.

**Santos, C., Rohde, K., Ramos, R., Di Beneditto, A. and Capistrano, L.** (1996). Helminths of cetaceans on the Southeastern coast of Brazil. *Journal of the Helminthological Society of Washington* **63**, 149–152.

**Savage, K. N., Burek‐Huntington, K., Wright, S. K., Bryan, A. L., Sheffield, G., Webber, M., Stimmelmayr, R., Tuomi, P., Delaney, M. A. and Walker, W.** (2021). Stejneger’s beaked whale strandings in Alaska, 1995–2020. *Marine Mammal Science* **37**, 843–869. doi: 10.1111/mms.12780.

**Schick, L., IJsseldijk, L. L., Grilo, M. L., Lakemeyer, J., Lehnert, K., Wohlsein, P., Ewers, C., Prenger-Berninghoff, E., Baumgärtner, W., Gröne, A., Kik, M. J. L. and Siebert, U.** (2020). Pathological Findings in White-Beaked Dolphins (Lagenorhynchus albirostris) and Atlantic White-Sided Dolphins (Lagenorhynchus acutus) From the South-Eastern North Sea. *Frontiers in Veterinary Science* **7**, 262. doi: 10.3389/fvets.2020.00262.

**Schumacher, U., Horny, H., Heidemann, G., Schultz, W. and Welsch, U.** (1990). Histopathological findings in harbour seals (Phoca vitulina) found dead on the german north sea coast. *Journal of Comparative Pathology* **102**, 299–309. doi: 10.1016/S0021-9975(08)80019-9.

**Seguel, M., Nadler, S., Field, C. and Duignan, P.** (2018). Vasculitis and Thrombosis due to the Sea Lion Lungworm, *Parafilaroides decorus* , in a Guadalupe Fur Seal ( *Arctocephalus philippii townsendi* ). *Journal of Wildlife Diseases* **54**, 638–641. doi: 10.7589/2017-12-291.

**Seguel, M., George, R., Maboni, G., Sanchez, S., Page-Karjian, A., Wirth, E., McFee, W. and Gottdenker, N.** (2020). Pathologic findings and causes of death in bottlenose dolphins Tursiops truncatus stranded along the Georgia coast, USA (2007-2013). *Diseases of Aquatic Organisms* **141**, 25–38. doi: 10.3354/dao03509.

**Seibel, H., Beineke, A. and Siebert, U.** (2010). Mycotic Otitis Media in a Harbour Porpoise (Phocoena phocoena). *Journal of Comparative Pathology* **143**, 294–296. doi: 10.1016/j.jcpa.2010.03.002.

**Sheldon, J. D., Johnson, S. P., Hernandez, J. A., Cray, C. and Stacy, N. I.** (2017). Acute-phase responses in healthy, malnourished, and Otostrongylus-infected juvenile northern elephant seals (mirounga angustirostris). *Journal of Zoo and Wildlife Medicine* **48**, 767–775. doi: 10.1638/2016-0267.1.

**Sheldon, J. D., Hernandez, J. A., Johnson, S. P., Field, C., Kaye, S. and Stacy, N. I.** (2019). Diagnostic Performance of Clinicopathological Analytes in Otostrongylus circumlitis-Infected Rehabilitating Juvenile Northern Elephant Seals (Mirounga angustirostris). *Frontiers in Veterinary Science* **6**, 134. doi: 10.3389/fvets.2019.00134.

**Shiozaki, A. and Amano, M.** (2017). Population- and growth-related differences in helminthic fauna of finless porpoises (Neophocaena asiaeorientalis) in five Japanese populations. *Journal of Veterinary Medical Science* **79**, 534–541. doi: 10.1292/jvms.16-0421.

**Siebert, U., Wünschmann, A., Weiss, R., Frank, H., Benke, H. and Frese, K.** (2001). Post-mortem Findings in Harbour Porpoises (Phocoena phocoena) from the German North and Baltic Seas. *Journal of Comparative Pathology* **124**, 102–114. doi: 10.1053/jcpa.2000.0436.

**Siebert, U., Tolley, K., Víkingsson, G. A., Ólafsdottir, D., Lehnert, K., Weiss, R. and Baumgärtner, W.** (2006). Pathological Findings in Harbour Porpoises (Phocoena phocoena) from Norwegian and Icelandic Waters. *Journal of Comparative Pathology* **134**, 134–142. doi: 10.1016/j.jcpa.2005.09.002.

**Siebert, U., Jepson, P. D. and Wohlsein, P.** (2013). First indication of gas embolism in a harbour porpoise (Phocoena phocoena) from German waters. *European Journal of Wildlife Research* **59**, 441–444. doi: 10.1007/s10344-013-0700-4.

**Siebert, U., Pawliczka, I., Benke, H., von Vietinghoff, V., Wolf, P., Pilāts, V., Kesselring, T., Lehnert, K., Prenger-Berninghoff, E., Galatius, A., Anker Kyhn, L., Teilmann, J., Hansen, M. S., Sonne, C. and Wohlsein, P.** (2020). Health assessment of harbour porpoises (PHOCOENA PHOCOENA) from Baltic area of Denmark, Germany, Poland and Latvia. *Environment International* **143**, 105904. doi: 10.1016/j.envint.2020.105904.

**Sierra, E., Zucca, D., Arbelo, M., García-Álvarez, N., Andrada, M., Déniz, S. and Fernández, A.** (2014). Fatal Systemic Morbillivirus Infection in Bottlenose Dolphin, Canary Islands, Spain. *Emerging Infectious Diseases* **20**, 269–271. doi: 10.3201/eid2002.131463.

**Smith, F. R. and Threlfall, W.** (1973). Helminths of Some Mammals from Newfoundland. *American Midland Naturalist* **90**, 215. doi: 10.2307/2424284.

**Stephens, N., Duignan, P. J., Wang, J., Bingham, J., Finn, H., Bejder, L., Patterson, A. P. and Holyoake, C.** (2014). Cetacean Morbillivirus in Coastal Indo-Pacific Bottlenose Dolphins, Western Australia. *Emerging Infectious Diseases* **20**, 672–676. doi: 10.3201/eid2004.131714.

**Stockin, K. A., Duignan, P. J., Roe, W. A., Meynier, L., Alley, M. and Fettermann, T.** (2009). Causes of mortality in stranded Common Dolphin (Delphinus sp.) from New Zealand waters between 1998 and 2008. *Pacific Conservation Biology* **15**, 217. doi: 10.1071/PC090217.

**Stroud, R. K.** (1978). Parasites and associated pathology observed in pinnipeds stranded along the Oregon coast. *Journal of Wildlife Diseases* **14**, 292–298. doi: 10.7589/0090-3558-14.3.292.

**Stroud, R. K. and Roffe, T. J.** (1979). Causes of death in marine mammals stranded along the oregon coast. *Journal of Wildlife Diseases* **15**, 91–97. doi: 10.7589/0090-3558-15.1.91.

**Suvorova, I. V. and Prokushina, K. S.** (2021). Pulmonary nematodiasis of the Baikal seal (Pusa siberica). In *Theory and practice of parasitic disease control: Collection of Scientific Articles adapted from the International Scientific Conference.*, pp. 509–514.

**Sweeney, J. C. and Gilmartin, W. G.** (1974). Survey of diseases in free-living California sea lions. *Journal of Wildlife Diseases* **10**, 370–376. doi: 10.7589/0090-3558-10.4.370.

**Szefer, P., Rokicki, J., Frelek, K., Skora, K. and Malinga, M.** (1998). Bioaccumulation of selected trace elements in lung nematodes, Pseudalius inﬂexus, of harbor porpoise (Phocoena phocoena) in a Polish zone of the Baltic Sea. *The Science of the Total Environment* **220**, 19–24.

**Tao, J.-Y.** (1983). A new species and a new chinese record of nematodes from porpoise neophocaenaphocaenoides. *Acta Zootaxonomica Sinica*.

**Terracciano, G., Fichi, G., Comentale, A., Ricci, E., Mancusi, C. and Perrucci, S.** (2020). Dolphins Stranded along the Tuscan Coastline (Central Italy) of the “Pelagos Sanctuary”: A Parasitological Investigation. *Pathogens* **9**, 612. doi: 10.3390/pathogens9080612.

**Tomilin, A.** (1967). Mammals of the USSR and adjacent countries, Vol. 9, Cetacea. *Israel program for scientific translations, Jerusalem* **71**,.

**Tomo, I., Kemper, C. M. and Lavery, T. J.** (2010). Eighteen-year study of south Australian dolphins shows variation in lung nematodes by season, year, age class, and location. *Journal of Wildlife Diseases* **46**, 488–498. doi: 10.7589/0090-3558-46.2.488.

**Torres, P., Cortes, P., Oporto, J. A., Brieva, L. and Silva, R.** (1994). The occurrence of stenurus australis Tantalean and Sarmiento, 1991(Nematoda: Mestratongyloidea)in the Porpoise Phocoena spinipinnis (Burmeister,1865) on the Southern Coast of Chile. *Memórias do Instituto Oswaldo Cruz* **89**, 141–143. doi: 10.1590/S0074-02761994000200004.

**Ulrich, S. A., Lehnert, K., Siebert, U. and Strube, C.** (2015). A recombinant antigen-based enzyme-linked immunosorbent assay (ELISA) for lungworm detection in seals. *Parasites & Vectors* **8**, 443. doi: 10.1186/s13071-015-1054-4.

**Ulrich, S. A., Lehnert, K., Rubio-Garcia, A., Sanchez-Contreras, G. J., Strube, C. and Siebert, U.** (2016). Lungworm seroprevalence in free-ranging harbour seals and molecular characterisation of marine mammal MSP. *International Journal for Parasitology: Parasites and Wildlife* **5**, 48–55. doi: 10.1016/j.ijppaw.2016.02.001.

**Valderrama Vasquez, C. A., Macgregor, S. K., Rowcliffe, J. M. and Jepson, P. D.** (2008). Occurrence of a monophasic strain of Salmonella group B isolated from cetaceans in England and Wales between 1990 and 2002. *Environmental Microbiology* **10**, 2462–2468. doi: 10.1111/j.1462-2920.2008.01651.x.

**van Elk, C. E., van de Bildt, M. W. G., van Run, P. R. W. A., Bunskoek, P., Meerbeek, J., Foster, G., Osterhaus, A. D. M. E. and Kuiken, T.** (2019). Clinical, pathological, and laboratory diagnoses of diseases of harbour porpoises (Phocoena phocoena), live stranded on the Dutch and adjacent coasts from 2003 to 2016. *Veterinary Research* **50**, 88. doi: 10.1186/s13567-019-0706-3.

**Vargas-Castro, I., Crespo-Picazo, J. L., Rivera-Arroyo, B., Sánchez, R., Marco-Cabedo, V., Jiménez-Martínez, M. Á., Fayos, M., Serdio, Á., García-Párraga, D. and Sánchez-Vizcaíno, J. M.** (2020). Alpha- and gammaherpesviruses in stranded striped dolphins (Stenella coeruleoalba) from Spain: first molecular detection of gammaherpesvirus infection in central nervous system of odontocetes. *BMC Veterinary Research* **16**, 288. doi: 10.1186/s12917-020-02511-3.

**Vercruysse, J., Salomez, A., Ulloa, A., Osterhaus, A., Kuiken, T. and Alvinerie, M.** (2003). Efficacy of ivermectin and moxidectin against Otostrongylus circumlitus and Parafilaroides gymnurus in harbour seals (Phoca vitulina). *Veterinary Record* **152**, 130–134. doi: 10.1136/vr.152.5.130.

**Veryeri, N. G.** (2012). Postmortem examinations of stranded dolphins found on the Black Sea coast near Ordu, Turkey (Mammalia: Cetacea). *Zoology in the Middle East* **55**, 129–132. doi: 10.1080/09397140.2012.10648928.

**von Waerebeek, K., Reyes, J. C. and Shigueto, J. A.** (1993). Helminth parasites and phoronts of dusky dolphins Lagenorhynchus obscurus (Gray, 1828) from Peru. *Aquatic Mammals* **19**, 159–169.

**Walden, H. D. S., Grijalva, C. J., Páez-Rosas, D. and Hernandez, J. A.** (2018). Intestinal Parasites in Galapagos Sea Lions (Zalophus wollebaeki) Sivertsen, 1953 on San Cristóbal Island, Galapagos, Ecuador. *Journal of Parasitology* **104**, 718–721. doi: 10.1645/17-187.

**Walden, H. S., Bryan, A. L., McIntosh, A., Tuomi, P., Hoover-Miller, A., Stimmelmayr, R. and Quakenbush, L.** (2020). Helminth fauna of ice seals in the Alaskan Bering and Chukchi seas, 2006–15. *Journal of Wildlife Diseases* **56**,. doi: 10.7589/2019-09-228.

**Walker, W. A.** (1975). Review of the Live-Capture Fishery for Smaller Cetaceans Taken in Southern California Waters for Public Display, 1966–73. *Journal of the Fisheries Research Board of Canada* **32**, 1197–1211. doi: 10.1139/f75-139.

**Wan, X., Zheng, J., Li, W., Zeng, X., Yang, J., Hao, Y. and Wang, D.** (2017). Parasitic infections in the East Asian finless porpoise Neophocaena asiaeorientalis sunameri living off the Chinese Yellow/Bohai Sea coast. *Diseases of Aquatic Organisms* **125**, 63–71. doi: 10.3354/dao03131.

**Wazura, K. W., Strong, J. T., Glenn, C. L. and Bush, A. O.** (1986). Helminths of the Beluga Whale (Delphinapterus leucas) from the Mackenzie River Delta, Northwest Territories. *Journal of Wildlife Diseases* **22**, 440–442. doi: 10.7589/0090-3558-22.3.440.

**Webster, W., Neufeld, J. and MacNeill, A.** (1973). Halocercus monoceris sp. n. (Nematoda: Metastrongyloidea) from the Narwhal, Monodon monoceros. *Proceedings of the Helminthological Society of Washington* **40**, 255–258.

**Williams, K. M., Fessler, M. K., Bloomfield, R. A., Sandke, W. D., Malekshahi, C. R., Keroack, C. D., Duignan, P. J., Torquato, S. D. and Williams, S. A.** (2020). A novel quantitative real-time PCR diagnostic assay for fecal and nasal swab detection of an otariid lungworm, Parafilaroides decorus. *International Journal for Parasitology: Parasites and Wildlife* **12**, 85–92. doi: 10.1016/j.ijppaw.2020.04.012.

**Wohlsein, P., Seibel, H., Beineke, A., Baumgärtner, W. and Siebert, U.** (2019). Morphological and Pathological Findings in the Middle and Inner Ears of Harbour Porpoises (Phocoena phocoena). *Journal of Comparative Pathology* **172**, 93–106. doi: 10.1016/j.jcpa.2019.09.005.

**Woodard, J. C., Zam, S. G., Caldwell, D. K. and Caldwell, M. C.** (1969). Some Parasitic Diseases of Dolphins. *Pathologia veterinaria* **6**, 257–272. doi: 10.1177/030098586900600307.

**Wu, H. W.** (1929). On Halocercus pingi n. sp. a Lung-Worm from the Porpoise, Neomeris phocoenoides. *The Journal of Parasitology* **15**, 276. doi: 10.2307/3271983.

**Wunschimann, A., Frese, K., Müiller, G., Baumgärtner, W., Siebert, U., Weiss, R., Lockyer, C. and Heide-Jørgensen, M. P.** (2001). Evidence of infectious diseases in harbour porpoises (Phocoena phocoena) hunted in the waters of Greenland and by-caught in the German North Sea and Baltic Sea. *Veterinary Record* **148**, 715–720. doi: 10.1136/vr.148.23.715.

**Wünschmann, A., Siebert, U. and Weiss, R.** (1999). Rhizopusmycosis in a Harbor Porpoise from the Baltic Sea. *Journal of Wildlife Diseases* **35**, 569–573. doi: 10.7589/0090-3558-35.3.569.

**Yamaguti, S.** (1951). Studies on the helminth fauna of Japan. Part 46. Nematodes of marine mammals. *Arbeiten aus der Medizinischen Fakultat Okayama* **7**, 295–306.

**Yu, J., Sun, Y. and Xia, Z.** (2009). The Rescue, Rehabilitation, and Release of a Stranded Finless Porpoise (Neophocaena phocaenoides sunameri) at Bohai Bay of China. *Aquatic Mammals* **35**, 220–225. doi: 10.1578/AM.35.2.2009.220.

**Zafra, R., Jaber, J. R., Pérez, J., de la Fuente, J., Arbelo, M., Andrada, M. and Fernández, A.** (2015). Immunohistochemical characterisation of parasitic pneumonias of dolphins stranded in the Canary Islands. *Research in Veterinary Science* **100**, 207–212. doi: 10.1016/j.rvsc.2015.03.021.

**Zam, S. G., Caldwell, D. K. and Caldwell, M. C.** (1971). Some endoparasites from small odontocete cetaceans collected in Florida and Georgia. *Cetology* **2**, 1–11.

**Zylber, M. I., Failla, G. and Le Bas, A.** (2002). Stenurus globicephalae Baylis et Daubney, 1925 (Nematoda: Pseudaliidae) from a False Killer Whale, Pseudorca crassidens (Cetacea: Delphinidae), Stranded on the Coast of Uruguay. *Memórias do Instituto Oswaldo Cruz* **97**, 221–225. doi: 10.1590/S0074-02762002000200015.
